# Structured 3′ UTRs destabilize mRNAs in plants

**DOI:** 10.1186/s13059-024-03186-x

**Published:** 2024-02-22

**Authors:** Tianru Zhang, Changhao Li, Jiaying Zhu, Yanjun Li, Zhiye Wang, Chun-Yip Tong, Yu Xi, Yi Han, Hisashi Koiwa, Xu Peng, Xiuren Zhang

**Affiliations:** 1 Department of Biochemistry and Biophysics, Texas A&M University, College Station, TX 77843 USA; 2 Molecular and Environmental Plant Sciences, Texas A&M University, College Station, TX 77843 USA; 3 State Key Laboratory for Managing Biotic and Chemical Threats to the Quality and Safety of Agro-products, Institute of Plant Virology, Ningbo University, Ningbo, 315211 China; 4 State Key Laboratory of Plant Physiology and Biochemistry, College of Life Sciences, Zhejiang University, Hangzhou, 310058 China; 5 Department of Medical Physiology, College of Medicine, Texas A&M University, Bryan, TX 77807 USA; 6 National Engineering Laboratory of Crop Stress Resistence Breeding, School of Life Sciences, Anhui Agricultural University, Hefei, 230036 China; 7 Department of Horticultural Sciences, Texas A&M University, College Station, TX 77843 USA; 8 Department of Biology, Texas A&M University, College Station, TX 77843 USA

**Keywords:** 3′ UTR, RNA secondary structure (RSS), 3′ end target-specific DMS-MaPseq, DIM-2P-seq, mRNA stability

## Abstract

**Background:**

RNA secondary structure (RSS) can influence the regulation of transcription, RNA processing, and protein synthesis, among other processes. 3′ untranslated regions (3′ UTRs) of mRNA also hold the key for many aspects of gene regulation. However, there are often contradictory results regarding the roles of RSS in 3′ UTRs in gene expression in different organisms and/or contexts.

**Results:**

Here, we incidentally observe that the primary substrate of miR159a (pri-miR159a), when embedded in a 3′ UTR, could promote mRNA accumulation. The enhanced expression is attributed to the earlier polyadenylation of the transcript within the hybrid pri-miR159a-3′ UTR and, resultantly, a poorly structured 3′ UTR. RNA decay assays indicate that poorly structured 3′ UTRs could promote mRNA stability, whereas highly structured 3′ UTRs destabilize mRNA in vivo. Genome-wide DMS-MaPseq also reveals the prevailing inverse relationship between 3′ UTRs’ RSS and transcript accumulation in the transcriptomes of *Arabidopsis*, rice, and even human. Mechanistically, transcripts with highly structured 3′ UTRs are preferentially degraded by 3′–5′ exoribonuclease SOV and 5′–3′ exoribonuclease XRN4, leading to decreased expression in *Arabidopsis*. Finally, we engineer different structured 3′ UTRs to an endogenous *FT* gene and alter the *FT*-regulated flowering time in *Arabidopsis*.

**Conclusions:**

We conclude that highly structured 3′ UTRs typically cause reduced accumulation of the harbored transcripts in *Arabidopsis*. This pattern extends to rice and even mammals. Furthermore, our study provides a new strategy of engineering the 3′ UTRs’ RSS to modify plant traits in agricultural production and mRNA stability in biotechnology.

**Supplementary Information:**

The online version contains supplementary material available at 10.1186/s13059-024-03186-x.

## Background

RNA possesses a sophisticated structure resulting from intramolecular or intermolecular base pairing. Genome-wide profiling of RSS implicates their functional links to the regulation of transcription and posttranscriptional processing. For example, the flexible regions have been often detected upstream of the 5′ splice sites, and so have the folded 3′ end region of mRNA in front of polyadenylation sites (poly(A) sites) [1, 2]. Furthermore, RSS can serve as a docking place for assembling ribonucleoprotein complexes (RNPs), which in turn participate in diverse functions [3]. Various types of RNA, such as tRNA and rRNA, rely on their unique structures to interpret the central dogma via translation [4, 5]. Riboswitches undergo conformational changes in response to altered levels of ligands to fine tune gene expression and translation of the mRNA [6, 7]. The dynamic nature of RSS makes it a quick whistle and pivotal mediator in response to environmental changes and functional needs [8]. Thus, precise study of RSS’ roles in determining mRNA fates will not only reveal new regulatory layers of biological processes, but also provide new targets in synthetic biology to improve agricultural traits or biotechnological products [9].

The 3′ UTR is a trailing stretch of mRNA and has been initially considered as an auxiliary and useless addition to an mRNA, but mounting evidence suggests 3′ UTRs are instrumental in several aspects of gene expression, influencing mRNA stability, protein synthesis, subcellular localization of RNPs, and ultimately protein functions [10–13]. Additionally, 3′ UTRs contain regulatory elements formed by secondary structures and have different effects on gene expression in diverse contexts [14]. In yeast, one class of such elements is a polyU sequence that can interact with poly(A) tails to form double-stranded (ds) RNA structure, and such structure inhibits the association of poly(A)-binding protein, leading to increased mRNA stability [14]. It has been also noticed that mRNA isoforms engineered to contain 3′ stem-loops tend to have longer half-lives, leading to a proposal that double-stranded structures at 3′ ends are a major determinant of mRNA stability [14]. In mammalian cells, genome-wide profiling of endogenous mRNAs reveals that the 3′ end region downstream poly(A) signal is more folded compared to elsewhere [2]. The folding of mRNA 3′ regions optimizes the distance between the poly(A) signal and the poly(A) sites, which facilitates efficient cleavage and polyadenylation to enhance mRNA stability [2]. Increasing mRNA stability by 3′ end folding is in line with the observation that some viral RNAs have evolved to contain the complicated secondary structures and exhibit increased stability, possibly by impeding the digestion of poly(A) tails by deadenylases [15]. One such structure is exemplified by the formation of a triple helix involving the poly(A) tail and other RNA elements in viral genes [15–17]. Paradoxically, another study shows that mRNAs with highly structured 3′ UTRs are easily subjected to RNA decay, while the poorly structured 3′ UTRs are more stable [18]. Altogether, there appear to be contradictory results regarding the roles of 3′ UTRs in gene expression in different organisms and/or contexts. How 3′ UTRs impact the fates of mRNAs is largely unknown in plants.

A group of small non-coding regulatory RNAs, microRNAs (miRNAs), play critical roles in numerous biological processes in eukaryotes. miRNA biogenesis requires Microprocessor, comprising Dicer-like 1 (DCL1) and two RNA binding proteins (RBPs), Hyponastic leaves 1 (HYL1), and Serrate (SE), to recognize the hairpin folded structure of pri-miRNAs and precisely process the substrates into miRNAs [19, 20]. We have recently adopted an idea to develop stable transgenic lines expressing pri-miRNA-embedded in 3′ UTR region of *luciferase* (*LUC*) [21] as a reporter to assess plant Microprocessor activity in vivo. To our big surprise, the inclusion of pri-miR159a in the 3′ UTR substantially increased mRNA expression, rather than decreasing it. The reason was that the reporter transcripts were earlier polyadenylated at a 5′ end opened-structured segment of pri-miR159a that turned out to increase the accumulation of the harbored transcript. We further expanded the study and comprehensively compared the effect of different 3′ UTRs on gene expression. We found that the presence of highly structured 3′ UTRs indeed caused the reduction of *LUC* transcripts, which was degraded by 3′–5′ exoribonuclease SUPPRESSOR OF VARICOSE (SOV) and 5′–3′ EXORIBONUCLEASE 4 (XRN4). Furthermore, the genome-wide analysis revealed an inverse association between the 3′ UTRs’ RSS and gene expression levels in diverse organisms. Subsequently, we genetically engineered RSS of 3′ UTR to manipulate the transgene expression of *FLOWERING LOCUS T* (*FT*), resulting in an earlier flowering phenotype in *Arabidopsis*. Thus, this study unveiled the negative regulatory role of 3′ UTRs’ RSS in gene expression and suggested a novel strategy for engineering RSS of 3′ UTRs to modulate traits in agricultural production.

## Results

### Insertion of *MIR159a* in 3′ UTR substantially increases the expression of transgene

**Fig. 1:**
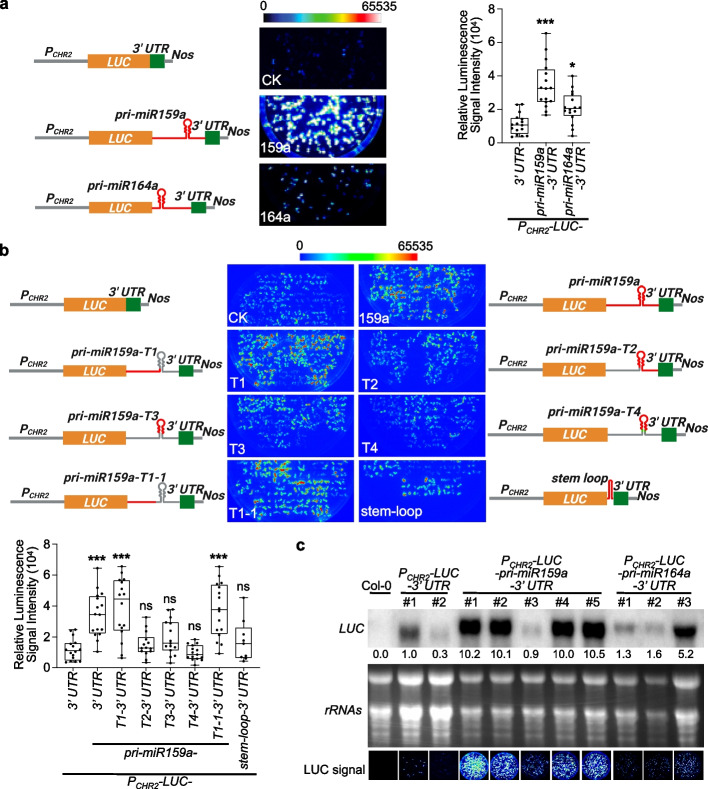
Insertion of *MIR159a* in 3′ UTR enhances the expression of transgene. **a**, **b** LUC signals of reporter lines expressing different constructs. (Left part) schematic constructs. *P*_*CHR2*_, the native promoter of *CHR2* locus; *Nos*, nopaline synthase terminator. For different truncated segments of pri-miR159a, the red lines represented the retained regions of pri-miR159a, while the gray regions were removed in the constructs. Be noted that pri-miR159a-T4, but not pri-miR159a-T3 contained the miR159/159* duplex (labeled in green). (Middle part) Six-day-old T2 seedlings of 16 randomly selected independent lines were photographed under charge-coupled device (CCD) camera for LUC signals. Exposure time for CCD camera was 30 S. (Right part in **a** and lower part in **b**) Quantification of luminescence results from different transgene lines. Each data point represented the mean of 10–12 plants from individual lines. For most constructs, 16 individual lines were utilized except for *P*_*CHR2*_*-LUC-stem-loop-3*′ *UTR* where only 9 lines were available. Whiskers represent the minimum and maximum values whereas horizontal lines in the boxplots display the 75^th^, 50^th^, and 25^th^ percentiles, respectively. Statistical test was performed between different transgenic lines and *P*_*CHR2*_*-LUC-3*′ *UTR*. ns, no significance; *P < 0.05; **P < 0.01; ***P < 0.001; unpaired two-tailed Student’s *t* test. **c** RNA blot analyses of randomly selected lines showed *LUC* transcripts significantly accumulated in *P*_*CHR2*_*-LUC-pri-miR159a-3*′ *UTR*, but not *P*_*CHR2*_*-LUC-pri-miR164a-3*′ *UTR*, compared to *P*_*CHR2*_*-LUC-3*′ *UTR*. Ribosomal RNAs served as control. LUC signals of the sampled materials were shown in the bottom panels (Exposure time of 30 S under CCD camera). The relative signals of *LUC* blot were first normalized to that of *rRNAs*, and then to that of #1 of *P*_*CHR2*_*-LUC-3*′ *UTR* sample where the ratio was arbitrarily assigned a value of 1.0. Be noted that LUC pictures in **a** and **c** were taken under a CCD camera (Olympus DP70) different from the one used in **b** (Schneider Kreuznach), with each experiment having its own CK (*P*_*CHR2*_*-LUC-3*′ *UTR*) lines

To assess Microprocessor activity in vivo, a *LUC* reporter that utilizes portions of pri-miRNAs embedded in the 3′ UTR of the Renilla *LUC* gene was previously designed [21]. Pri-miRNAs are featured with a hairpin folded structure flanked by 5′ and 3′ open segments. The principle was that cleavage by Microprocessor is expected to destabilize the *LUC* mRNA and lead to decreased luminescence. Such a reporter system has been shown to serve as a sensitive readout of Microprocessor activity in mammalian cells in a transient expression system [21]. Recently, we have tried to adopt the idea to monitor plant Microprocessor activity in vivo. Specifically, we constructed pri-miR159a and pri-miR164a, which produce two founding members of plant miRNAs, miR159, and miR164, respectively, onto the downstream of a *LUC* gene. Such fusion construct is flanked by the promoter and 3′ UTR elements of an endogenous gene, *chromatin-remodeling factor 2*, *CHR2* (*AT2G46020*), and transformed into *Arabidopsis* to create stable transgenic plants (*P*_*CHR2*_*-LUC-pri-miR159a-3*′ *UTR* and *P*_*CHR2*_*-LUC-pri-miR164a-3*′ *UTR*). We found that the inclusion of pri-miR159a in the 3′ UTR substantially increased the accumulation of *LUC* mRNA and luminescence, by more than 10- and three-fold compared to the control construct, respectively, in which pri-miR159a was deleted (CK, *P*_*CHR2*_*-LUC-3*′ *UTR*) (Fig. 1a–c). In contrast, the insertion of pri-miR164a into the 3′ UTR could only have negligible or marginal effects on the luminescence signal (Fig. 1a, c). Given the positional and dosage effects of transgenes, we randomly selected at least 16 independent lines for each construct and measured LUC activities (Fig. 1a, b). The profiling of the LUC activities in the large population clearly indicated that the insertion of the pri-miR159a into the 3′ UTR could significantly enhance the expression of transgenes (Fig. 1a, b).

To investigate if the impact of the hybrid pri-miR159a-3′ UTR on the expression of the harbored transgene was accredited to the activity of plant Microprocessor, we introduced the chimeric construct *P*_*CHR2*_*-LUC-pri-miR159a-3*′ *UTR* into *dcl1-9* (+ / −) and *se-3* (+ / −), respectively. Among T2 populations, *dcl1-9* and *se-3* homozygotes (− / −) displayed smaller statues and obvious developmental defects compared to the heterozygotes and wild-type siblings, and thus easily discerned. Notably, *dcl1-9* (− / −) and *se-3* (− / −), which are defective in pri-miRNA-processing, showed no significant difference in LUC signals compared to that in the heterozygotes (+ / −) and wild-type siblings (Additional file 1: Fig. S1a). These findings suggested that pri-miR159a embedded within the chimeric construct was not responsive to Microprocessor defects. Alternatively, pri-miR159a when inserted in the 3′ UTR was not expressed.

To pinpoint the causative segment of pri-miR159a in enhancing LUC signal, we generated four truncated segments of pri-miR159a (T1, 1–274 nucleotide (nt), comprising the 5′ segment; T2, 275–619 nt, consisting of the stem-loop structure and 3′ bases; T3, 305–444 nt, representing a partial stem-loop without miR159/159* duplex; T4, 275–476 nt, containing the stem-loop and miR159/159* duplex) (Fig. 1b, schematics). CCD imaging revealed that the stable transgenic plants expressing the 5′ end segment of pri-miR159a (1–274 nt) (159a-T1, *P*_*CHR2*_*-LUC-pri-miR159a-T1-3*′ *UTR*) displayed comparable LUC intensity to the ones in the lines with full-length pri-miR159a, whereas the lines expressing other truncations (159a-T2/T3/T4) including the pre-miRNA or the 3′ bases of pri-miR159a did not (Fig. 1b). These results suggested that the elevated LUC signal of *P*_*CHR2*_*-LUC-pri-miR159a-3*′ *UTR* was not attributable to the self-folded pre-miRNA structure but rather to unidentified elements in the 5′ end segment of pri-miR159a.

To further clarify the effect of the self-folded structure, we purposely introduced a new stem-loop in the 3′ UTR (*P*_*CHR2*_*-LUC-stem-loop-3*′ *UTR*). In contrast to the inclusion of pri-miR159a-T1, introducing an artificial stem-loop segment in the 3′ UTR had marginal or varied effects on the LUC signal vs *P*_*CHR2*_*-LUC-3*′ *UTR* (Fig. 1b). This result again suggested that some new features of the hybrid pri-miR159a-3′ UTR might confer the accumulation of LUC signal in *P*_*CHR2*_*-LUC-pri-miR159a-3*′ *UTR*. To test this, we first determined the poly(A) site for the *LUC* transcript of *P*_*CHR2*_*-LUC-pri-miR159a-3*′ *UTR* by performing 3′ rapid amplification of cDNA ends (3′ RACE). It turned out that the poly(A) site was located right before the stem-loop structure of pri-miR159a (Additional file 1: Fig. S1b). This result explained why *P*_*CHR2*_*-LUC-pri-miR159a-3*′ *UTR* and *P*_*CHR2*_*-LUC-pri-miR159a-T1-3*′ *UTR* transgenic lines had comparable LUC signals. The 3′ RACE result also explained why the chimeric construct was not responsive to Microprocessor activity (Additional file 1: Fig. S1a). Subsequently, we further trimmed pri-miR159a-T1 and found that pri-miR159a-T1-1 transgenic plants (*P*_*CHR2*_*-LUC-pri-miR159a-T1-1-3*′ *UTR*; T1-1, 1–211 nt) exhibited a comparable LUC signal intensity to those observed in *P*_*CHR2*_*-LUC-pri-miR159a-3*′ *UTR* and *P*_*CHR2*_*-LUC-pri-miR159a-T1-3*′ *UTR* (Fig. 1b). Altogether, these results indicated that a novel element or segment within the 5′ end sequence of pri-miR159a, but not the hairpin-structured region, could enhance *LUC* expression when it was inserted into the 3′ UTR.

### Enhancement of transgene expression by pri-miR159a-T1-3′ UTR is not due to miPEPs or transcriptional change

In addition to serving as resources for miRNAs, some plant pri-miRNAs have been reported to contain short open reading frame (ORF) sequences that encode regulatory peptides (miRNA-encoded peptides, miPEPs). These short peptides can promote pri-miRNA transcription and miRNA accumulation [22]. According to this criterion, pri-miR159a-T1 contains two ORFs that might encode hypothetical 17-amino acid (AA) and 7-AA peptides (Additional file 1: Fig. S1c). To determine whether these hypothetical peptides might potentially promote the accumulation of steady-state *LUC* mRNA, we introduced missense mutations (ATG to TTG) and obtained transgenic plants with the mutated constructs. We observed that the double-mutation lines (*P*_*CHR2*_*-LUC-pri-miR159a-T1-DM-3*′ *UTR*) did not show compromised luminescence compared to the lines expressing *P*_*CHR2*_*-LUC-pri-miR159a-T1-3*′ *UTR* (Additional file 1: Fig. S1c). These results indicated that the enhanced LUC activity by pri-miR159a-T1 did not result from the presence of hypothetical small peptides.

Next, we assessed whether the fusion of pri-miR159a/159a-T1 in the 3′ UTR affected Pol II transcription efficiency, thereby increasing *LUC* mRNA and protein levels. We performed Pol II-ChIP-qPCR to measure the relative abundance of RNA Pol II at the different loci of the *LUC* locus. Our findings showed that Pol II abundance was higher at the *LUC* region than the *CHR2* promoter region, but there was no significant difference in Pol II abundance onto the same positions among transgenic lines with different LUC levels (low LUC, *P*_*CHR2*_*-LUC-3*′ *UTR*, and high LUC, *P*_*CHR2*_*-LUC-pri-miR159a-3*′ *UTR* and *P*_*CHR2*_*-LUC-pri-miR159a-T1-3*′ *UTR*) (Additional file 1: Fig. S1d). These results indicated that the enhanced LUC signals in *P*_*CHR2*_*-LUC-pri-miR159a-3*′ *UTR* and *P*_*CHR2*_*-LUC-pri-miR159a-T1-3*′ *UTR* were not attributed to increased transcription efficiency. Therefore, the results suggested that a novel mechanism(s) might account for the enhanced LUC signals by the pri-miR159a/159a-T1-embedded 3′ UTR.

### 3′ end target-specific DMS-MaPseq shows that *LUC* transgene expression is inversely related to RSS of 3′ UTRs

**Fig. 2:**
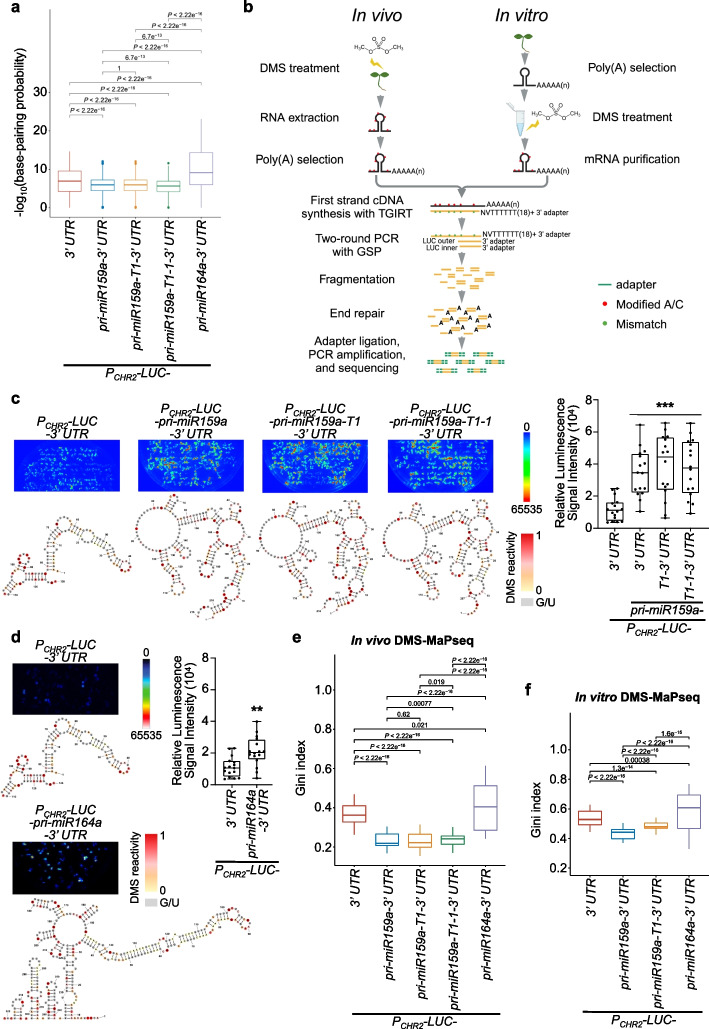
*LUC* transgene expression is inversely correlated with RSS of 3′ UTRs. **a** Predicted base-pairing probabilities of the 3' UTRs for different transgene lines (*P*_*CHR2*_*-LUC-3*′ *UTR*, *P*_*CHR2*_*-LUC-pri-miR159a-3*′ *UTR*, *P*_*CHR2*_*-LUC-pri-miR159a-T1-3*′ *UTR*, *P*_*CHR2*_*-LUC-pri-miR159a-T1-1-3*′ *UTR*, and *P*_*CHR2*_*-LUC-pri-miR164a-3*′ *UTR*) via RNAstructure [23]. *P* values by Wilcoxon test. **b** Schematic pipeline of 3′ end target-specific DMS-MaPseq for both in vivo and in vitro conditions. See Methods for details. GSP, gene specific primer; TGIRT, thermostable group II intron reverse transcriptase. **c**, **d** RSS of the 3′ UTRs for different transgene transcripts in **a**. The DMS signals of A and C residues were color-coded and U/G bases were marked in gray. Quantification of luminescence results of the representative samples was shown in the right part. LUC pictures in **c** and **d** were taken under different CCD cameras, with each experiment having its own CK (*P*_*CHR2*_*-LUC-3*′ *UTR*) lines. Whiskers represent the minimum and maximum values whereas horizontal lines in the boxplots display the 75^th^, 50^th^, and 25^th^ percentiles, respectively. Statistical test was performed between different transgenic lines and *P*_*CHR2*_*-LUC-3*′ *UTR*. ns, no significance; *P < 0.05; **P < 0.01; ***P < 0.001; unpaired two-tailed Student’s *t* test. **e**–**f** Gini index of in vivo (**e**) and in vitro (**f**) DMS reactivities of the 3′ UTRs for different transgene lines. *P* values by Wilcoxon test. In **a**, **e**, and **f**, horizontal lines in the boxplots display the 75^th^, 50^th^, and 25^th^ percentiles, respectively. The upper fence is 75^th^ percentile + 1.5 * interquartile range. The lower fence is 25^th^ percentile − 1.5 * interquartile range. Dots represent the outliers

We next hypothesized that RSS of 3′ UTR might cause increased LUC signals in *P*_*CHR2*_*-LUC-pri-miR159a-3*′ *UTR* and *P*_*CHR2*_*-LUC-pri-miR159a-T1-3*′ *UTR* lines. In light of the similar enhancement of LUC signals in *P*_*CHR2*_*-LUC-pri-miR159a-3*′ *UTR*, *P*_*CHR2*_*-LUC-pri-miR159a-T1-3*′ *UTR*, and *P*_*CHR2*_*-LUC-pri-miR159a-T1-1-3*′ *UTR* compared to *P*_*CHR2*_*-LUC-3*′ *UTR* and *P*_*CHR2*_*-LUC-pri-miR164a-3*′ *UTR*, we conducted 3′ RACE experiment of these transgenic lines to identify their poly(A) sites. *P*_*CHR2*_*-LUC-pri-miR164a-3*′ *UTR* and *P*_*CHR2*_*-LUC-3*′ *UTR* had their poly(A) sites in the 5′ end or the middle of the 3′ UTR region. Differently, *P*_*CHR2*_*-LUC-pri-miR159a-3*′ *UTR*, *P*_*CHR2*_*-LUC-pri-miR159a-T1-3*′ *UTR*, and *P*_*CHR2*_*-LUC-pri-miR159a-T1-1-3*′ *UTR* lines all had their poly(A) sites at the 5′ end segment of pri-miR159a, rather than extending through the hairpin-structure region to reach the very 3′ end of their 3′ UTRs (Additional file 1: Fig. S1b). Interestingly, both *P*_*CHR2*_*-LUC-pri-miR159a-3*′ *UTR* and *P*_*CHR2*_*-LUC-pri-miR159a-T1-3*′ *UTR* exhibited poly(A) sites at the same position, specifically within the pri-miR159a-T1 region. Similarly, *P*_*CHR2*_*-LUC-pri-miR159a-T1-1-3*′ *UTR* was also polyadenylated at a nearby site (Additional file 1: Fig. S1b). We then employed RNAstructure [23] to predict the base-pairing probability of RNA sequences of their “3′ UTR” regions based on their poly(A) sites. We found that the low *LUC* expression lines (*P*_*CHR2*_*-LUC-3*′ *UTR* and *P*_*CHR2*_*-LUC-pri-miR164a-3*′ *UTR*) exhibited higher base-pairing probabilities of 3′ UTRs compared to the high *LUC* expression lines (*P*_*CHR2*_*-LUC-pri-miR159a-3*′ *UTR*, *P*_*CHR2*_*-LUC-pri-miR159a-T1-3*′ *UTR*, and* P*_*CHR2*_*-LUC-pri-miR159a-T1-1-3*′ *UTR*). This implied that *P*_*CHR2*_*-LUC-3*′ *UTR* and *P*_*CHR2*_*-LUC-pri-miR164a-3*′ *UTR* may possess more-paired RSS of 3′ UTRs in comparison to the high *LUC* expression lines (Fig. 2a).

To test whether RSS of 3′ UTRs might impact the accumulation of transgene transcripts, we adapted DMS mutational profiling with Illumina sequencing (DMS-MaPseq) strategy and performed 3′ end DMS-MaPseq to specifically target 3′ UTRs of *LUC* in vivo [2, 24, 25]. Briefly, DMS reacts with unpaired adenosines (As) and cystines (Cs) in RNA, preventing their proper pairing from the complementary strands in reverse transcription (RT). In DMS-MaPseq, the thermostable group II intron reverse transcriptase (TGIRT) decodes DMS lesions on RNA templates as mismatches on cDNA [24, 25]. For this experiment, one DMS-untreated reference sample and three DMS-treated samples were used for library construction. Polyadenylated mRNA was isolated, followed by RT via TGIRT. The cDNA was subjected to two-round PCR with LUC-specific primer to enrich target *LUC* products. The resultant products were then adaptered and sequenced (Fig. 2b left part). The DMS reactivities among three biological replicates were highly reproducible with the lowest Pearson’s correlation coefficient being 0.7562, and the majority of coefficients being more than 0.9 (Additional file 1: Fig. S2a). Nucleotide modifications in DMS-treated samples exhibited high specificity towards As and Cs (Additional file 1: Fig. S2b). Additionally, we verified the reliability of our in vivo 3′ end target-specific DMS-MaPseq strategy by amplifying *CAB1* mRNA and mapping its DMS reactivities, which exhibited strong agreement with previously published data (PCC = 0.626) (Additional file 1: Fig. S2c) [26]. These results showed that our 3′ end target-specific DMS-MaPseq could accurately probe RSS in vivo.

Next, we assessed the structural features of RSS in 3′ UTRs across different transgenic lines using the Gini index, a metric that measures structural heterogeneity along a transcript. A higher Gini index signifies a more structured RSS whereas a lower Gini index refers to a less structured RSS [27]. To quantify the structural differences, we employed a sliding-window method [24] to compute the Gini index for each construct (details in “Methods”). Similar to the base-pairing probability of RSS (Fig. 2a), the 3′ UTRs from the high *LUC* expression lines (*P*_*CHR2*_*-LUC-pri-miR159a-3*′ *UTR*, *P*_*CHR2*_*-LUC-pri-miR159a-T1-3*′ *UTR*, and* P*_*CHR2*_*-LUC-pri-miR159a-T1-1–3*′ *UTR*) had significantly lower Gini index than the ones of the low *LUC* expression lines (*P*_*CHR2*_*-LUC-3*′ *UTR* and *P*_*CHR2*_*-LUC-pri-miR164a-3*′ *UTR*), indicating that the 3′ UTRs of the formers were less structured compared to the latter ones (Fig. 2c–e). Specifically, *P*_*CHR2*_*-LUC-3*′ *UTR* displayed a small three-way junction with a long stem-loop structure. Similarly, *P*_*CHR2*_*-LUC-pri-miR164a-3*′ *UTR* had a big six-way junction but with two compact clusters of short stem-loops and a long stem-loop structure. By contrast, *P*_*CHR2*_*-LUC-pri-miR159a-3*′ *UTR*, *P*_*CHR2*_*-LUC-pri-miR159a-T1-3*′ *UTR*, and* P*_*CHR2*_*-LUC-pri-miR159a-T1-1-3*′ *UTR* all displayed at multiple-junction structures with big loops and a few short stem-loops (Fig. 2c, d).

Some studies suggest that in vivo low DMS reactivities might result from protein footprinting of RNA [28], whereas others do not [29]. To clarify if the highly structured features in the 3′ UTRs of the low *LUC* expression lines observed from in vivo DMS-MaPseq were due to protein binding, we conducted in vitro 3′ end target-specific DMS-MaPseq (Fig. 2b right part) for the isolated but in vitro refolded RNA from selected low and high *LUC* expression lines. Remarkably, we still observed significantly lower Gini index of the 3′ UTRs of *LUC* transcripts from the high expression lines (*P*_*CHR2*_*-LUC-pri-miR159a-3*′ *UTR* and *P*_*CHR2*_*-LUC-pri-miR159a-T1-3*′ *UTR*) than the ones from the two low *LUC* expression lines (*P*_*CHR2*_*-LUC-3*′ *UTR* and* P*_*CHR2*_*-LUC-pri-miR164a-3*′ *UTR*), reminiscent of in vivo 3′ end target-specific DMS-MaPseq results (Fig. 2e, f). Thus, the higher Gini index observed in the 3′ UTRs of *LUC* transcripts in the low expression lines in vivo are due to their intrinsic RSS, rather than the hindrance of DMS modifications by protein binding. Together, these findings suggested that RNA transcripts with highly structured 3′ UTRs might have lower gene expression, while transcripts with poorly structured 3′ UTRs could accumulate to a higher level.

Transgenic lines of *P*_*CHR2*_*-LUC-pri-miR159a-T2-3*′ *UTR* displayed low *LUC* expression, likely due to the presence of the intrinsic hairpin structure (Fig. 1b; Additional file 1: Fig. S3b). We wondered if the disruption of the structure would impact the LUC signal. To this end, we constructed two more truncations (T2-1, 277–309 nt; T2-2, 429–554 nt) of pri-miR159a-T2 and generated transgenic lines (*P*_*CHR2*_*-LUC-pri-miR159a-T2-1-3*′ *UTR* and *P*_*CHR2*_*-LUC-pri-miR159a-T2-2-3*′ *UTR*). Interestingly, different from *P*_*CHR2*_*-LUC-pri-miR159a-T2-3*′ *UTR* and *P*_*CHR2*_*-LUC-stem-loop-3*′ *UTR* lines that exhibited comparable LUC signals with *P*_*CHR2*_*-LUC-3*′ *UTR*, the two pri-miR159a-T2 truncation lines (*P*_*CHR2*_*-LUC-pri-miR159a-T2-1-3*′ *UTR* and *P*_*CHR2*_*-LUC-pri-miR159a-T2-2-3*′ *UTR*) displayed significantly enhanced LUC signals, reminiscent of *P*_*CHR2*_*-LUC-pri-miR159a-3*′ *UTR* and *P*_*CHR2*_*-LUC-pri-miR159a-T1-3*′ *UTR* lines (Additional file 1: Fig. S3b). Subsequently, we identified their poly(A) sites (Additional file 1: Fig. S3a) and modeled their RSS of “3′ UTR” region using RNAStructure software [23]. Again, the RSS of 3′ UTRs in the low *LUC* expression lines (*P*_*CHR2*_*-LUC-pri-miR159a-T2-3*′ *UTR* and *P*_*CHR2*_*-LUC-stem-loop-3*′ *UTR*) were more paired/structured, similar to that of *P*_*CHR2*_*-LUC-3*′ *UTR*, whereas the RSS of the high *LUC* expression lines (*P*_*CHR2*_*-LUC-pri-miR159a-T2-1-3*′ *UTR* and *P*_*CHR2*_*-LUC-pri-miR159a-T2-2-3*′ *UTR*) were more single-stranded (Additional file 1: Fig. S3b). We further assessed the base-pairing probability for individual lines and observed that the low *LUC* expression lines (*P*_*CHR2*_*-LUC-3*′ *UTR*, *P*_*CHR2*_*-LUC-pri-miR159a-T2-3*′ *UTR*, and *P*_*CHR2*_*-LUC-stem-loop-3*′ *UTR*) exhibited significantly higher base-pairing probabilities in their 3′ UTRs compared to the high *LUC* expression lines (*P*_*CHR2*_*-LUC-pri-miR159a-T2-1-3*′ *UTR* and *P*_*CHR2*_*-LUC-pri-miR159a-T2-2-3*′ *UTR*) (Additional file 1: Fig. S3c). Collectively, our results suggested that the transgene expression of *LUC* is inversely correlated to the RSS of 3′ UTRs.

### Transcripts with poorly structured 3′ UTR exhibit high RNA stability both in vivo and in vitro

**Fig. 3:**
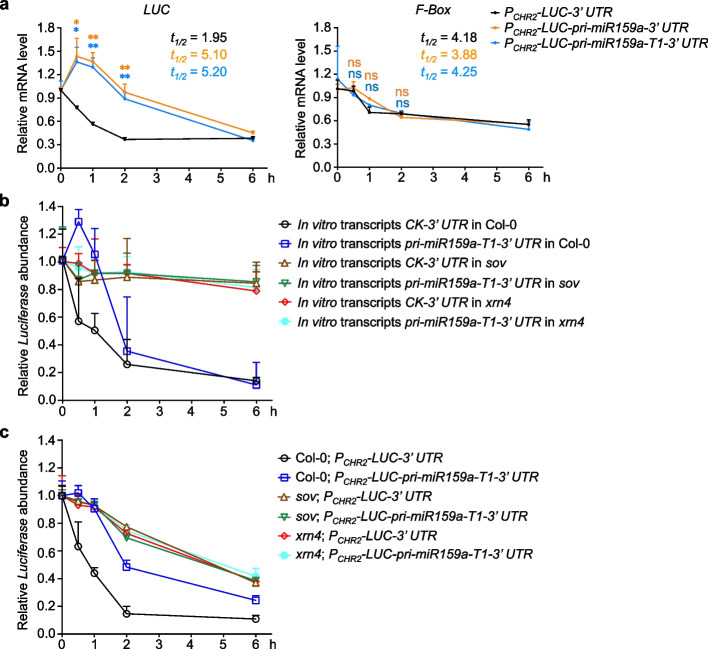
Poorly structured 3′ UTRs increase RNA stability to enhance transcript accumulation. **a** qRT-PCR assays showed the relative expression of *LUC* (left) and *F-box* (right) in different transgenic samples (*P*_*CHR2*_*-LUC-3*′ *UTR*, *P*_*CHR2*_*-LUC-pri-miR159a-3*′ *UTR*, and *P*_*CHR2*_*-LUC-pri-miR159a-T1-3*′ *UTR*) collected at indicated times after the treatment with 50 μM Act D. Half-life *t*_1/2_ (h) is shown. *LUC*_*CK*_* t*_1/2_ < *LUC*_*159a/159a-T1*_* t*_1/2_. *F-box*_*CK*_* t*_1/2_ ≅ *F-box*_*159a/159a-T1*_* t*_1/2_. RNA was extracted from 10-day-old seedlings of each line. The relative expression of tested genes was normalized to that of *18S rRNA*. **b** qRT-PCR showed the relative mRNA abundance of in vitro transcribed 3′ end region of *LUC* transcripts of *P*_*CHR2*_*-LUC-3*′ *UTR* (*CK-3*′ *UTR*) and *P*_*CHR2*_*-LUC-pri-miR159a-T1-3*′ *UTR* (*pri-miR159a-T1-3*′ *UTR*) delivered into Col-0, *sov*, and *xrn4*. The same level of in vitro transcripts was infiltrated into 10-day-old Col-0, *xrn4*, and *sov* seedlings, respectively. Infiltrated plants were collected at indicated time points for qRT-PCR. A different in vitro transcribed segment of *LUC* transcript was co-infiltrated as a reference for normalization. **c** qRT-PCR showed that the decay of *LUC* transcripts with highly structured 3′ UTRs is through SOV and XRN4. Ten-day-old seedlings were treated with 50 μM Act D for indicated times before sampling. The relative expression of *LUC* was normalized to that of *18S rRNA*. Data from **a**–**c** are shown as means ± SE from three independent biological replicates. ns, no significance; *P < 0.05; **P < 0.01; unpaired two-tailed Student’s *t* test

We next hypothesized that the RSS in the 3′ UTRs may affect the stability of transcripts. To test this, we treated transgenic plants with Actinomycin D (Act D), a chemical that inhibits transcription activities of RNA polymerase I, II, and III [30], and then determined the *LUC* mRNA half-life. While the endogenous transcript of *F-Box* (AT2G18780) showed similar decay rates in all kinds of transgenic lines, we observed significantly slower decay rates of *LUC* transcripts in *P*_*CHR2*_*-LUC-pri-miR159a-3*′ *UTR* and *P*_*CHR2*_*-LUC-pri-miR159a-T1-3*′ *UTR* compared to that of *P*_*CHR2*_*-LUC-3*′ *UTR* (Fig. 3a). These results indicated that poorly structured 3′ UTRs of *P*_*CHR2*_*-LUC-pri-miR159a-3*′ *UTR* and *P*_*CHR2*_*-LUC-pri-miR159a-T1-3*′ *UTR* indeed enhanced *LUC* mRNA stability.

To determine whether the 3′ UTR was enough for triggering structure-mediated RNA decay (SRD) [18], we performed in vitro transcription of the 3′ end regions of *P*_*CHR2*_*-LUC-3*′ *UTR* and *P*_*CHR2*_*-LUC-pri-miR159a-T1-3*′ *UTR*. We then infiltrated the same amount of in vitro transcripts into 10-day-old Col-0 seedlings and measured their RNA decay rates. Again, *pri-miR159a-T1-3*′ *UTR* transcript exhibited a slower decay rate than the one of *CK-3*′ *UTR* (Fig. 3b, in Col-0), further indicating that the highly structured 3′ UTR alone is sufficient to promote the SRD pathway.

In the cytoplasm, most mRNAs are subjected to deadenylation, followed by either 3′–5′ decay through exosome or exoribonuclease SOV, or 5′–3′ decay, which involves decapping and exoribonucleolytic decay by EXORIBONUCLEASE (XRN1 or XRN4). To investigate whether SRD depended on the cytoplasmic exoribonuclease activities, we infiltrated the aforementioned transcripts into *xrn4* and *sov* mutants. qRT-PCR assays revealed that the overall decay of both *CK-3*′ *UTR* and *pri-miR159a-T1-3*′ *UTR* transcripts slowed down in the mutants (*xrn4* and *sov*) vs Col-0, suggesting that the infiltrated transcripts could be degraded through these two pathways. Additionally, the higher decay rate of *CK-3*′ *UTR* transcript was abolished, and the two kinds of in vitro transcripts displayed similar decay patterns in the mutants (*xrn4* and *sov*) vs Col-0 (Fig. 3b).

We next assessed the *LUC* transcripts decay rate in the stable transgenic lines. To this end, we crossed *P*_*CHR2*_*-LUC-3*′ *UTR* and *P*_*CHR2*_*-LUC-pri-miR159a-T1-3*′ *UTR* lines with *sov* and *xrn4* mutants, respectively. The segregation in F2 population allowed us to concurrently obtain the stable lines of *P*_*CHR2*_*-LUC-3*′ *UTR* and *P*_*CHR2*_*-LUC-pri-miR159a-T1-3*′ *UTR* in the mutants (*sov* and *xrn4*) and Col-0 backgrounds. Again, the overall decay rates of *LUC* transcripts in all lines slowed down in mutants (*xrn4* and *sov*) compared to that of Col-0 (Fig. 3c). Furthermore, the significant difference between *P*_*CHR2*_*-LUC-3*′ *UTR* and *P*_*CHR2*_*-LUC-pri-miR159a-T1-3*′ *UTR* lines observed in Col-0 background now disappeared in the mutants. In addition, *LUC* transcripts from two transgenic lines displayed similar decay patterns in the mutants (*xrn4* and *sov*) vs Col-0, alike to the results from the semi in vitro assays (Fig. 3b, c). Collectively, we concluded that the less structured 3′ UTRs become more resistant to the activities of SOV and XRN4, leading to the accumulation of *LUC* in *P*_*CHR2*_*-LUC-pri-miR159a-T1-3*′ *UTR* vs *P*_*CHR2*_*-LUC-3*′ *UTR* line.

### Genome-wide structure data exhibits an inverse relationship between 3′ UTRs’ RSS and gene expression across different organisms

**Fig. 4:**
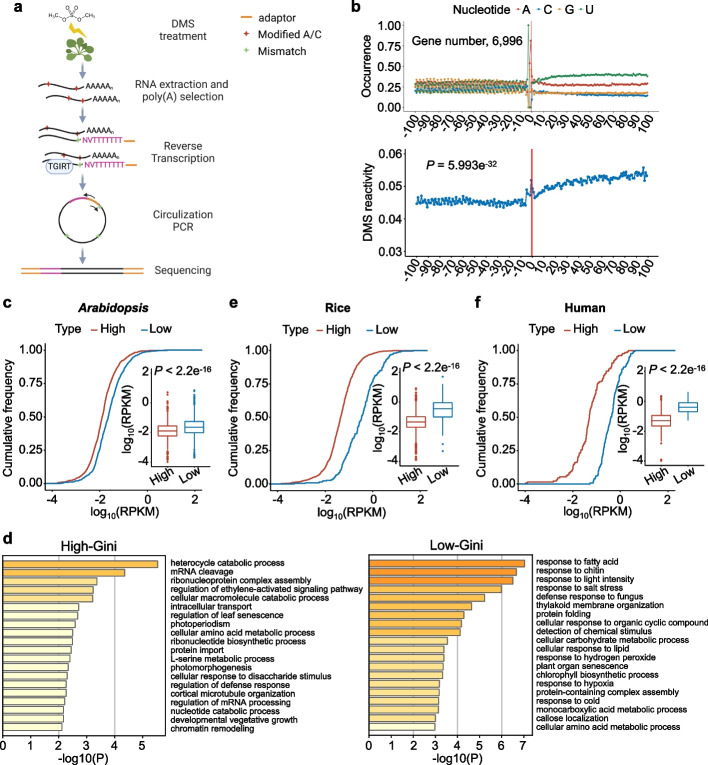
3′ UTRs’ RSS is inversely related to transcript accumulation in *Arabidopsis*, rice, and human. **a** Schematic of DIM-2P-seq used for in vivo probing 3′ end RSS of polyadenylated transcripts. See “Methods” for details. **b** DMS reactivity profile of stop codon regions (lower panel, CDS, 100 nt upstream of the stop codon, and 3′ UTR, 100 nt downstream of the stop codon). mRNAs were aligned by their stop codons (vertical red lines). 6996 genes were used in this analysis.* P* value = 5.993e^−32^ by Wilcoxon test between DMS reactivities of CDS and 3′ UTR. Nucleotide frequency around the stop codon regions was also shown (upper panel). **c**, **e**, **f** Comparison of gene expression level (RPKM) between the high-Gini and low-Gini genes for *Arabidopsis* (**c**), rice (**e**), and human (**f**). *P* value < 2.2e^−16^ by Wilcoxon test. Horizontal lines in the boxplots display the 75^th^, 50^th^, and 25^th^ percentiles, respectively. The upper fence is 75^th^ percentile + 1.5 * interquartile range. The lower fence is 25^th^ percentile − 1.5 * interquartile range. Dots represent the outliers. **d** Biological processes of GO analysis for the high-Gini and low-Gini genes. *P* value cutoff, 0.01

To investigate whether the inverse relationship between RSS of 3′ UTRs and gene expression obtained from the *LUC* reporter lines was also applicable to the entire transcriptome of *Arabidopsis*, we utilized DMS-induced mutations mapped by 2P-seq (DIM-2P-seq, Fig. 4a) to measure the RSS of 3′ UTRs globally [2]. Briefly, Col-0 plants were treated with or without DMS, followed by isolation of polyadenylated mRNA. The mRNA was subsequently fragmented via partial digestion using ribonuclease T1, which exhibits specificity in cleaving after guanine residues. The resultant fragments, containing a poly(A) tail, were purified, and subjected to RT via TGIRT, using a primer specifically designed to anneal to the beginning of the poly(A) tail. Finally, the cDNA was sequenced (Fig. 4a). The high reproducibility of three DMS-treated replicates is shown in Additional file 1: Fig. S4a, c, d. We observed that the reads for DIM-2P-seq data were enriched at the 3′ end (Additional file 1: Fig. S4b left) compared to regular RNA-seq in which sequencing reads were primarily distributed through the coding sequence (CDS) regions (Additional file 1: Fig. S4b right). The RSS modeling of U1 snRNA and *CAB1* from our DIM-2P-seq was consistent with previous studies (Additional file 1: Fig. S4e) [26, 31]. These results indicated that our DIM-2P-seq possessed a high sequencing quality, enabling us to investigate the RSS of endogenous 3′ UTRs in vivo.

Subsequently, we analyzed DMS reactivities of transcript segments 100 nt upstream and downstream of stop codons from 6996 mRNAs. Notably, the DMS reactivities of the 3′ UTR were significantly higher than those of CDS (*P* value = 5.993e^−32^), possibly due to the low GC content in the 3′ UTR (Fig. 4b). This finding is consistent with previous studies that demonstrated a higher degree of base pairing in the CDS compared to the 3′ UTR [32, 33]. Moreover, the codon periodicity was absent in the 3′ UTR, which was characteristic in the CDS (see “Methods” for details; Additional file 1: Fig. S4f). This pattern difference may be attributed to the translation activity of ribosomes as shown in previous studies [26, 32].

We next classified the transcriptome into three categories according to Gini index: the 3′ UTRs with the 10% highest Gini index (highly structured, 905 genes); the 3′ UTRs with the 10% lowest Gini index (poorly structured, 904 genes), and the rest in the middle. We mined our previously reported RNA-seq data obtained from the same experimental condition [34]. Then, we compared expression levels of the genes with the top 10% of high-Gini values with the ones that had the bottom 10% of low-Gini values (see “Methods” for details). We found that the expression levels of the low-Gini transcripts were significantly higher than those of the high-Gini transcripts (Fig. 4c). The negative correlation between RSS of 3′ UTRs (Gini index ) and transcript expression levels (RPKM) was also supported by a scatter plot of correlation analysis (*R* = − 0.12 and *P* < 2.22e^−16^; Additional file 1: Fig. S5a). These results indicated that the inverse correlation between RSS of 3′ UTRs and transcript accumulation was widely present in *Arabidopsis* transcriptome. Moreover, GO enrichment analysis of the high-Gini and low-Gini genes from *Arabidopsis* revealed that the low-Gini genes are more likely to be involved in stress-related biological processes, such as response to fatty acid, response to chitin, response to light intensity, response to salt stress, etc. (Fig. 4d), indicative of their greater ability to change RNA conformations in response to external stimuli. Overall, our findings demonstrated that RNA transcripts with poorly structured 3′ UTRs displayed higher expression levels relative to those with highly structured 3′ UTRs, and these genes are involved in stress-related pathways.

We next investigated whether this inverse correlation between RSS of 3′ UTRs and gene expression went beyond *Arabidopsis*. To this end, we mined published datasets of RSS and RNA-seq from rice and human [35, 36]. Likewise, we determined the genes with the top 10% and the bottom 10% Gini index as the high-Gini and low-Gini genes in rice, reflecting 1247 (all have Gini index of 1) and 129 genes, respectively. Similarly, we obtained 129 genes for the high-Gini and low-Gini ones each for human. Interestingly, comparative studies showed that the high-Gini genes indeed maintained lower expression levels than the low-Gini genes both in rice and human (Fig. 4e, f; Additional file 1: Fig. S5b, c), reminiscent of the scenario in *Arabidopsis*. These results indicated that the inverse relationship between RSS of 3′ UTRs and gene expression is conserved across different organisms.

### Reverse relationship between RSS of 3′ UTRs and transcript accumulation is not confounded by selected sequence features

Considering that some sequence features, such as GC content [37–39], poly(A) tail length [40], and 3′ UTR length [41], regulate the transcript accumulation, we sought to determine whether these factors would confound the reverse relationship between RSS of 3′ UTRs and gene expression levels. Firstly, we explored the potential correlation between Gini index and GC content. Overall speaking, the genes with high-Gini 3′ UTRs exhibited lower GC content than the ones with low-Gini 3′ UTRs (Additional file 1: Fig. S5d left). However, this generic correlation mainly exists in the genes with exceedingly low GC content (< 32.55%, median; Additional file 1: Fig. S5d bottom middle). When focusing on the high-GC genes (GC content ≥ 32.55%), we did not observe any difference in GC content between the high-Gini and low-Gini genes (Additional file 1: Fig. S5d top middle). Nevertheless, for these high-GC genes, the high-Gini genes still had lower expression levels than the low-Gini genes, indicating that GC content of the 3′ UTRs in this setting does not seem to be a confounding factor for the correlation between Gini index and gene expression (Additional file 1: Fig. S5d top right).

Akin to GC content, the genes with high-Gini 3′ UTRs tended to have longer poly(A) tails than the ones with low-Gini 3′ UTRs (Additional file 1: Fig. S5e left), aligning with an earlier report that the transcripts with longer poly(A) tails are less stable in *Arabidopsis* [40]. When the genes were re-sorted into the long and short poly(A) tailed groups (poly(A) tail length ≥ or < 80 nt, median length), respectively, the positive correlation between Gini index and poly(A) tail length disappeared (possibly due to variability existing in genes with moderate poly(A) tail length) (Additional file 1: Fig. S5e middle). Nevertheless, we still observed that the high-Gini genes exhibited lower expression levels regardless of poly(A) tail lengths in either long or short poly(A) tailed groups (Additional file 1: Fig. S5e right).

Since long 3′ UTR was reported to destabilize mRNA, we subsequently assessed potential difference in the length of 3′ UTRs between the high-Gini and low-Gini genes [41]. Our results revealed no significant difference in the length of 3′ UTRs between the high-Gini and low-Gini genes (Additional file 1: Fig. S5f). Additionally, we investigated whether the identified high-Gini or low-Gini genes were associated with RNA G-quadruplex (RG4) structures in 3′ UTRs, which are known to enhance mRNA stability [42]. To this end, we employed “quadparser” software [43] to predict a total of 2722 RG4 sites based on *Arabidopsis* Araport11 genome [44]. Comparison of RG4 sites of 3′ UTRs between the high-Gini and low-Gini genes showed that only two high-Gini genes and one low-Gini gene 3′ UTRs possessed RG4 structures (Additional file 1: Fig. S5g), indicating that the mechanism by which the low-Gini genes displayed increased expression levels was not related to RG4 structure. In metazoan, miRNAs typically target 3′ UTRs of transcripts and trigger translational repression followed by RNA decay [45]. However, in plants, miRNAs can target the entire transcript body from 5′ UTR, ORFs to 3′ UTRs. This notwithstanding, we did not observe a significant difference in the distribution of miRNA-targeting sites between the high-Gini and low-Gini genes (Additional file 1: Fig. S5h). In summary, the identified negative correlation between RSS of 3′ UTRs and RNA expression levels is not confounded by the above-tested factors such as GC content, poly(A) tail length, 3′ UTR length, RG4, or miRNA targeting.

### Transcripts with poorly structured 3′ UTRs are more stable than the ones with highly structured 3′ UTRs

**Fig. 5:**
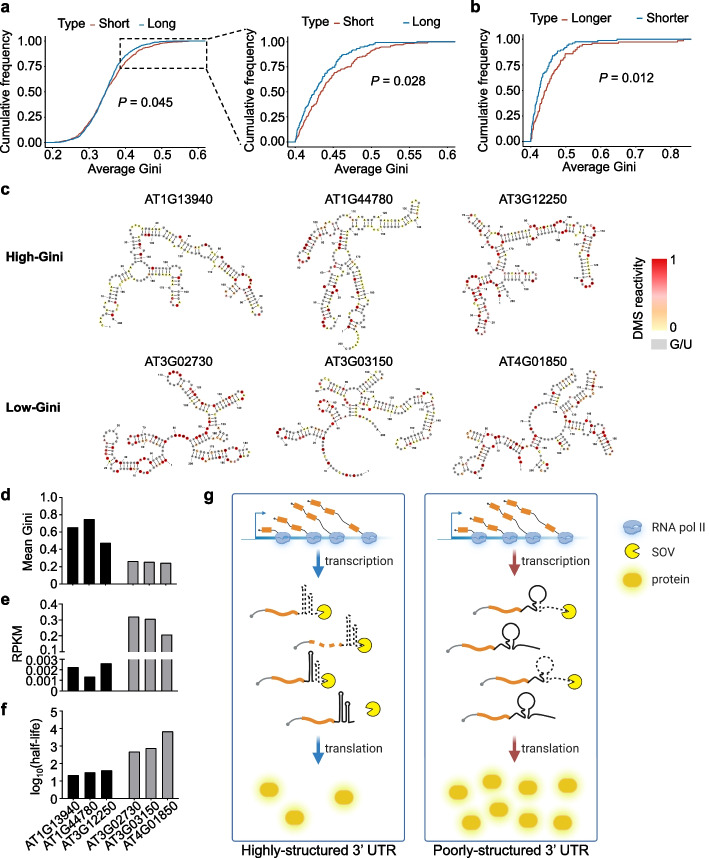
Transcripts with poorly structured 3′ UTR are more stable in *Arabidopsis*. **a** Comparison of Gini index between the short half-life and long half-life genes in WT for *Arabidopsis* (RNA decay data, GSE86361). *P* value by Kolmogorov-Smirnov test. **b** Comparison of RSS of 3′ UTRs (Gini index) between the longer half-life (the top 10% of log_2_(*sov*/WT)) and shorter half-life (the bottom 10% of log_2_(*sov*/WT)) genes in *sov* compared to WT in *Arabidopsis* (RNA decay data, GSE86361). *P* value by Kolmogorov-Smirnov test. **c** RSS modeling for randomly selected transcripts with the high-Gini and low-Gini 3′ UTRs. The DMS signals of A and C residues were color-coded and U/G bases were marked in gray. **d**–**f** Mean Gini index (**d**), expression level (RPKM) (**e**), and half-life (log_10_(min)) (**f**) of the gene examples in **c**. **g** A proposed model for RSS of 3′ UTR in regulating gene expression. This model shows that RNA transcripts possessing highly structured 3′ UTR are susceptible to degradation by 3′–5′ exoribonuclease SOV. Conversely, transcripts with less structured 3′ UTR could evade from the degradation, thereby exhibiting enhanced stability and expression

Since highly structured 3′ UTRs triggered quicker decay of transcripts in our reporter experiment, we wondered whether the low expression of genome-wide high-Gini genes would also result from their reduced RNA stability. To test this, we utilized publicly available RNA decay RNA-seq data to examine the relationship between RSS of 3′ UTRs and RNA stability [46]. In this case, we classified the transcripts into three categories based on their half-life values: the top 10% long half-life mRNAs (134 genes), the bottom 10% short half-life mRNAs (178 genes), and the rest in the middle. Then we calculated their Gini index, respectively. Interestingly, we discovered that the short half-life genes had significantly higher Gini index than the long half-life genes, especially when the Gini index were larger than about 0.4 (Fig. 5a). To validate this finding, we referred to an independent RNA decay dataset and observed a similar pattern [47]. After setting the Gini index threshold to 0.4, the short half-life genes were more structured than the long half-life genes, indicating that once the RSS reached a certain threshold, it would trigger RNA degradation (Additional file 1: Fig S6a). The conclusion was further substantiated by scatter plots of correlation analysis between RSS of 3′ UTRs (average Gini) and RNA stability (half-life) (Additional file 1: Fig. S6b, c), where an increase in the Gini index threshold from ≥ 0.3 to ≥ 0.4 notably improves Pearson’s correlation coefficient *R* value.

To examine if SOV was involved in the genome-wide decay of highly structured RNA transcripts, we compared published RNA half-life data from Col-0 and *sov* mutant [47]. The results showed that the transcripts with substantially longer half-lives (the top 10% of *sov*/WT, 78 genes) in *sov* exhibit significantly higher Gini index compared to those with shorter half-lives (the bottom 10% of *sov*/WT, 87 genes) in *sov* (Fig. 5b). This intriguing result meaned that SOV may preferentially bind to the transcripts with highly structured 3′ UTRs in vivo. Essentially, this finding suggested that SOV is implicated in the decay of more structured transcripts as observed in the reporter assays (Fig. 5b; Additional file 1: Fig. S6d).

Furthermore, we randomly selected three representative transcripts with high and low Gini index, respectively, and modeled the RSS of 3′ UTRs of the transcripts. Again, the high-Gini transcripts exhibited more-paired RSS of 3′ UTRs compared to the low-Gini mRNAs (Fig. 5c). Moreover, the high-Gini genes had relatively low expression levels and short half-lives (Fig. 5d–f). Notably, all these three low-Gini genes are involved in stress-related pathways, which further implies that the genes with poorly structured 3′ UTRs are associated with stress responses (Figs. 4d and 5c) [48–50]. Taken together, our genome-wide sequencing analysis also revealed an inverse association between RSS of 3′ UTRs and mRNA stability, as observed in the *LUC* reporter lines. The studies also strongly suggested that SOV is involved in the decay of highly structured transcripts in *Arabidopsis*.

In summary, this suggests that the structural features of the 3′ UTR can play a crucial role in the regulation of RNA stability and gene expression. The extent of structural complexity in the 3′ UTR of RNA transcripts could affect their susceptibility to degradation by specific exoribonucleases, such as SOV. RNA transcripts with more complex and structured 3′ UTRs are more likely to be degraded, while those with less structured 3′ UTRs are more stable and exhibit higher expression levels (Fig. 5g).

### Addition of a poorly structured 3′ UTR to *FT* promotes early flowering phenotype

**Fig. 6:**
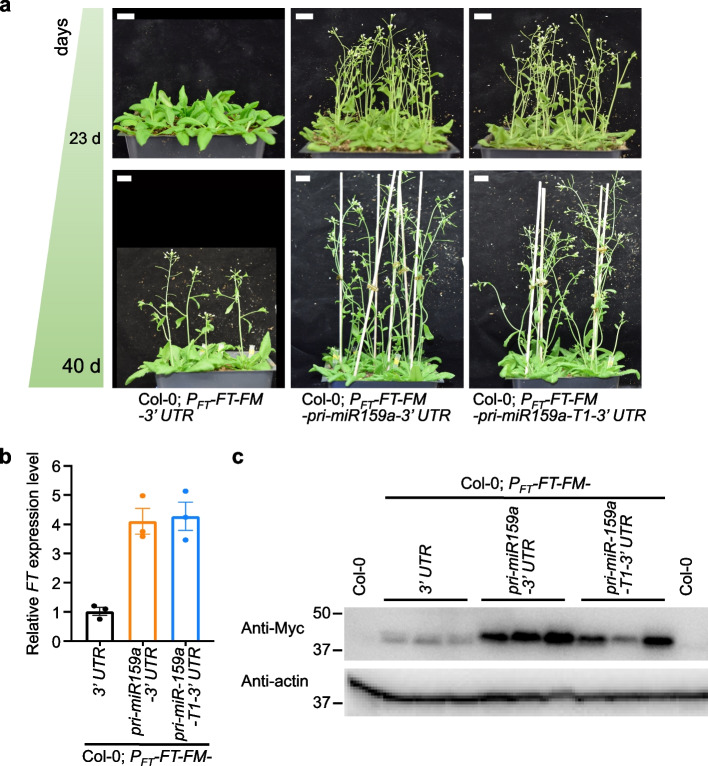
Engineered poorly structured 3′ UTRs of *FT* induce early flowering. **a** Flowering phenotype of the indicated stages in different T2 transformants with selected poorly structured or highly structured 3′ UTRs in Col-0 background. At least three individual lines were employed for each transgenic construct to observe the flowering phenotype, with 40–50 plants cultivated for each individual line in two independent sets of experiments (upper and lower panels). Consistent results were obtained across the two independent sets. Plants were grown under long-day (LD, 16 h: 8 h, light: dark) photoperiod conditions. Scale bar, 2 cm. **b** Relative expression of *FT* levels in T2 transgenic lines. The data were presented as means ± SE (*n* = 3) biologically independent replicates. The relative *FT* expression was normalized to that of *UBQ10*. **c** Western blot analysis showed increased FT protein levels in T2 transgenic lines with the poorly structured 3′ UTRs (Col-0; *P*_*FT*_*-FT-FM-pri-miR159a-3*′ *UTR* and Col-0; *P*_*FT*_*-FT-FM-pri-miR159a-T1-3*′ *UTR*) vs the highly structured ones (Col-0; *P*_*FT*_*-FT-FM-3*′ *UTR*). Anti-Myc antibody was used to detect Myc-tagged FT protein. Col-0 samples served as negative controls. Actin was a loading control

We next wondered if we could engineer RSS of 3′ UTRs to alter mRNA accumulation and plant traits. To this end, we modified the 3′ UTR of a flowering-related gene, *FT*, that positively promotes flowering [51]. We fused *FT* with several 3′ UTR fragments harboring different RSS forms and transformed them into Col-0. In T2 generation, we employed at least three individual lines for each transgenic construct to investigate their impact on flowering phenotype, with each individual line growing 40–50 plants. Indeed, the transgenic plants carrying poorly structured 3′ UTRs (*P*_*FT*_*-FT-Flag-4Myc(FM)-pri-miR159a-3*′ *UTR* and* P*_*FT*_*-FT-FM-pri-miR159a-T1-3*′ *UTR*) exhibited an earlier flowering phenotype compared to those carrying highly structured 3′ UTR (*P*_*FT*_*-FT-FM-3*′ *UTR*) (Fig. 6a). Importantly, qRT-PCR and western blot assays revealed that *FT* transcripts and FT protein accumulated much higher in the early flowering transgenic lines, *P*_*FT*_*-FT-FM-pri-miR159a-3*′ *UTR* and *P*_*FT*_*-FT-FM-pri-miR159a-T1-3*′ *UTR*, than that in *P*_*FT*_*-FT-FM-3*′ *UTR* line (Fig. 6b, c). These results clearly demonstrated that engineering poorly structured 3′ UTRs could increase *FT* gene expression and promote flowering, with a further suggestion that RSS of 3′ UTRs could be utilized in synthetic biology to control gene expression.

## Discussion

While studying miRNA biogenesis, we incidentally found that the *LUC* reporter expression is enhanced by the presence of pri-miR159a within the 3′ UTR (Fig. 1). The expression increment is attributed to the earlier polyadenylation within the hybrid pri-miR159a-3′ UTR and resultantly, a poorly structured 3′ UTR appending to the reporter ORF (Fig. 2; Additional file 1: Fig. S3). Genome-wide 3′ end structurome analysis also reveals the globally inverse relationship between RSS of 3′ UTRs and gene expression. More specifically, the more linearized 3′ UTRs the RNAs possess, the higher expression the transcripts show. On the other hand, the more RSS of 3′ UTRs, the lower accumulation of the mRNAs (Fig. 4). RNA decay analysis shows that the more double-stranded the RNA is, the shorter half-life it has, suggesting that decreased decay rate of transcripts contributes to the increased expression of RNAs with poorly structured 3′ UTRs (Figs. 3 and 5; Additional file 1: Fig. S6). Remarkably, the reciprocal association between 3′ UTRs’ RSS and gene expression is not limited to *Arabidopsis* but is also observed in rice and human, as demonstrated by analyzing several previously published datasets (Fig. 4; Additional file 1: Fig. S5a-c). Taken together, our findings support a model that structured 3′ UTRs can reduce gene expression via triggering RNA decay in vivo.

The 3′ UTR has been identified as the primary regulator of gene expression, as compared to the 5′ UTR or CDS region, due to several key factors. Firstly, the 3′ UTR is typically longer than the 5′ UTR, providing a larger platform for the binding of regulatory factors, including RBPs, miRNAs, and other non-coding RNAs [10, 52, 53]. The increased length also allows for more extensive alternative polyadenylation, leading to greater transcript diversity and regulatory potential. Secondly, the 3′ UTR is less constrained by ribosomal engagement compared to the CDS regions, which are typically occupied by ribosomes during translation [53]. This allows for more flexibility in the regulatory functions. Additionally, the 3′ UTR may contain regulatory elements, such as AU-rich elements, which were initially considered to be mRNA decay elements [13, 54, 55]. RBPs that recognize AU-rich elements facilitate deadenylation and exoribonuclease degradation [56, 57]. In zebrafish, 3′ UTR sequence elements, including AU-rich, CCUC, and CUGC elements regulate mRNA stability during maternal-to-zygotic transition [58]. We searched for these motifs in our high-Gini and low-Gini genes and did not find any motif bias within these two categories, which excluded the possibility that the known sequence elements could contribute to the stability difference between the high-Gini and low-Gini genes (data not shown). Additional search of potential new motifs via MEME recovered U-rich and A-rich motifs in the high-Gini 3′ UTRs, while CU-rich and U-rich motifs in the low-Gini 3′ UTRs [59]. Whether these motifs are *bona fide* elements and/or related to gene expression awaits future clarification (Additional file 1: Fig. S6e).

Notably, in our study, transcripts bearing diverse 3′ UTRs exhibited distinct poly(A) sites, a phenomenon potentially attributed to distinct poly(A) signals inherent to their respective 3′ UTR regions. In contrast to mammals, the conservation of poly(A) signals in plants is notably less pronounced [60]. Despite of our deliberate omission of the canonical poly(A) signal hexamer A(A/U)UAAA, it appears that certain sequences within the 3′ UTRs might function as poly(A) signals, thereby eliciting premature poly(A) sites. In addition, it has been documented that the structural features of DNA and RNA, leading to RNA Pol II pausing, can significantly contribute to the preferential utilization of specific poly(A) sites [61].

How does 3′ UTRs’ RSS affect RNA stability? We envisaged that 3′ UTRs’ RSS regulation is likely through yet unidentified RNPs that can recognize the RSS and channel the harbored transcripts to ribonuclease for degradation. It has been reported in other organisms that the structured 3′ UTRs, especially, the structure between the poly(A) signals and the poly(A) sites, can stabilize RNA [2]. However, others also reported that the overall highly structured 3′ UTRs can destabilize RNA, which is through UPF1 and G3BP1 [18]. It appears that our results in plants are in favor of the latter model rather than the former one. However, whether 3′ UTRs in plants undergo UPF1/G3BP1-mediated mechanism to regulate expression waits for future clarification. It has been reported that RNA expression is lower when the RNA structure is unfolded by heat shock in plants [62]. Another earlier report showed that heat-induced RNA decay is mediated by XRN4 [63]. One proposal was that the structure melting in the UTRs makes the transcripts easily targeted by 3′–5′ exoribonuclease such as SOV or 5′–3′ exoribonuclease such as XRN1/4. However, our global 3′ end RSS profiling and RNA decay data revealed that the poorly structured 3′ UTRs make the RNA transcripts more stable than those with highly structured 3′ UTRs (Fig. 5). To compromise the inconsistence, we proposed that the gene expression regulated through 3′ UTRs’ RSS is mechanistically distinct from the one regulated by heat-induced unfolding and degradation of RNA. Alternatively, the heat-induced RNA decay might be through 5′ UTRs and decapping mechanism.

It does not escape our attention that the decay rate of *LUC* transcripts from both constructs (*P*_*CHR2*_*-LUC-3*′ *UTR* and *P*_*CHR2*_*-LUC-pri-miR159a-T1-3*′ *UTR*) is similar in *sov* and *xrn4* mutants (Fig. 3). The results suggest that the highly structured 3′ UTRs may direct the transcripts to either 3′–5′ exoribonuclease pathway by SOV or 5′–3′ exoribonuclease pathway by XRN4. Although we did not directly prove SOV or XRN4 prefers to bind structured RNA, our transgenic *LUC* reporter assay and bioinformatic analysis provide a strong hint that SOV favors the decay of transcripts with highly structured 3′ UTRs (Figs. 3 and 5b). To our best knowledge, it has not been shown that SOV prefers to degrade dsRNAs or ssRNAs [47, 64]. A recent study found that dsRNA could trigger the change of protein conformation of Dis3L2, a human homolog of plant SOV, which in turn promotes structured RNA degradation [65]. Our results, together with a previous report that showed the substrate of SOV overlapped with decapping complex [64], suggest a critical role of 3′ UTRs in regulating RNA decay from both 3′–5′ and 5′–3′.

In summary, we found that poorly structured 3′ UTR stabilizes RNA in *Arabidopsis* (Fig. 5). Importantly this pattern seems to be conserved in eukaryotes [18]. Notably, it has been reported that 3′ UTR variants are often associated with human traits and diseases via GWAS analysis and computational prediction in human [66, 67]. A similar scenario has been also reported in plants [68]. The functional difference of these RiboSNitches within 3′ UTRs has been proposed to be related to the poly(A) site selection [67]. However, our current discovery might provide an alternative interpretation to these phenomena. That is, some of the 3′ UTR variants might modify RSS to fine tune the accumulation levels, resulting in physiological defects in human and plants. Importantly, the fact that the poorly structured 3′ UTRs stabilize RNA whereas highly structured 3′ UTRs lead to RNA destabilization provides a new idea to control gene expression in synthetic biology. The advantage of this new strategy is that the changes in 3′ UTRs can fine tune transcripts’ accumulation without any mutagenesis in protein coding sequence would largely facilitate genetic modification of crops. We also think that such a strategy can also be adopted in the field of vaccine production to enhance RNA stability and production. At the technical level, our 3′ end DMS-MaPseq data complement the shortcomings of the existing high-throughput structural probing assays, which miss structures at the very 3′ ends of RNAs. Thus, the 3′ end RSS profiling strategy, along with the traditional high-throughput structural probing method, provides a comprehensive overview of RSS of the transcriptome in *Arabidopsis* and beyond.

## Conclusions

In this study, we incidentally discovered that the pri-miR159a when embedded in the 3′ UTR of *CHR2* could promote the accumulation of the harbored transcript (Fig. 1), and this effect is not related to Microprocessor processing, Pol II transcription, or miPEPs function (Additional file 1: Fig. S1). The enhanced luminescence is attributed to the earlier polyadenylation within the hybrid pri-miR159a-3′ UTR and, resultantly, a poorly structured 3′ UTR appending to the reporter ORF (Fig. 2; Additional file 1: Fig. S3). We found that the poorly structured 3′ UTRs could promote mRNA stability, leading to the accumulation of the transcripts whereas highly structured 3′ UTR destabilizes the mRNA in vivo (Fig. 3). We extended this specific reporter gene to a genome-wide survey of transcriptome by performing DIM-2P-seq and discovered the prevailing inverse relationship between 3′ UTRs’ RSS and transcript accumulation in the whole transcriptome of *Arabidopsis*. Interestingly, the pattern that the genes with highly structured 3′ UTRs have lower expression levels and shorter half-lives than those with poorly structured 3′ UTRs can also extend to the transcriptomes of rice and even human (Fig. 4). Mechanistically, transcripts with highly structured 3′ UTRs are preferentially degraded by 3′–5′ exoribonuclease SOV, leading to decreased gene expression levels (Figs. 3 and 5). Finally, we engineered different structured 3′ UTRs to an endogenous *FT* gene and were able to alter the *FT*-regulated flowering time in *Arabidopsis* (Fig. 6). Thus, our study provides a new strategy by engineering the 3′ UTRs’ RSS to modify plant traits in agricultural production and mRNA stability in biotechnology.

## Methods

### Plant materials and growth conditions

*Arabidopsis thaliana* ecotype Columbia (Col-0), *se-3* (SALK_083196), *dcl1-9* (CS3828), *xrn4-6* (salk_014209), and *sov* (salk_017934) were used for this study. To generate transgenic lines, binary vectors containing *P*_*CHR2*_*-LUC-FM-3*′ *UTR*, *P*_*FT*_*-FT-FM-3*′ *UTR*, or their derivatives were transformed into the Col-0 ecotype of *A. thaliana* by the floral dip transformation method [69]. Transgenic plants were screened by western blot analysis or LUC assays. The *se-3; P*_*CHR2*_*-LUC-FM-pri-miR159a-3*′ *UTR* and *dcl1-9; P*_*CHR2*_*-LUC-FM-pri-miR159a-3*′ *UTR* materials were obtained by crossing the *P*_*CHR2*_*-LUC-FM-pri-miR159a-3*′ *UTR* with *se-3* (+ / −) or *dcl1-9* (+ / −), respectively. Homozygous *se-3*; *P*_*CHR2*_*-LUC-FM-pri-miR159a-3*′ *UTR* and *dcl1-9*; *P*_*CHR2*_*-LUC-FM-pri-miR159a-3*′ *UTR* were identified in the F2 generation by phenotyping and genotyping using primers listed in Additional file 2: Table S1. The *sov*;* P*_*CHR2*_*-LUC-FM-3*′ *UTR*, *sov*;* P*_*CHR2*_*-LUC-FM-pri-miR159a-3*′ *UTR*, *xrn4*;* P*_*CHR2*_*-LUC-FM-3*′ *UTR*, and *xrn4*;* P*_*CHR2*_*-LUC-FM-pri-miR159a-3*′ *UTR* materials were obtained by crossing *P*_*CHR2*_*-LUC-FM-3*′ *UTR* and *P*_*CHR2*_*-LUC-FM-pri-miR159a-3*′ *UTR* with *sov* and *xrn4*, respectively. Homozygotes of *sov*;* P*_*CHR2*_*-LUC-FM-3*′ *UTR*, *sov*;* P*_*CHR2*_*-LUC-FM-pri-miR159a-3*′ *UTR*, *xrn4*;* P*_*CHR2*_*-LUC-FM-3*′ *UTR*, and *xrn4*;* P*_*CHR2*_*-LUC-FM-pri-miR159a-3*′ *UTR*, and Col-0;* P*_*CHR2*_*-LUC-FM-3*′ *UTR* and Col-0;* P*_*CHR2*_*-LUC-FM-pri-miR159a-3*′ *UTR* materials were identified in the F2 generation by genotyping using primers listed in Additional file 2: Table S1. All plants were grown at 22 °C in 12-h light/12-h dark photoperiod (unless otherwise noted) on soil or MS plates as previously described [70].

### Vector construction and transgenic plants

Most of the constructs were generated by a Gateway cloning system (Invitrogen) and cloned into pENTR vectors. All constructs were confirmed by sequencing. The primers used for all constructs are listed in Additional file 2: Table S1.

The pBA002a-P_CHR2_-DC-FM-3′ UTR was described previously [19]. The LUC fragment was transferred into the binary vector of pBA002a-P_CHR2_-DC-FM-3′ UTR by Gateway attL-attR (LR) Clonase (Invitrogen) to obtain pBA002a-P_CHR2_-LUC-FM-3′ UTR. Pri-miR164a, pri-miR159a, and its truncation fragments were amplified by KOD Hot Start polymerase (Novagen) from Col-0 genomic DNA. Then, XhoI/SpeI-digested fragments were ligated into XhoI/SpeI-digested P_CHR2_-LUC-FM-3′ UTR to obtain pBA002a-P_CHR2_-LUC-FM-pri-miR164a-3′ UTR, pBA002a-P_CHR2_-LUC-FM-pri-miR159a-3′ UTR, and their derivatives.

To obtain pBA002a-P_CHR2_-LUC-FM-stem-loop-3′ UTR, P_CHR2_-LUC-FM-3′ UTR was digested with XhoI and SpeI, and the ends were blunted using DNA Polymerase I Large (Klenow) Fragment (NEB). Then, the artificial stem loop fragment was digested with ScaI, and the resultant fragments were ligated to generate pBA002a-P_CHR2_-LUC-FM-stem-loop-3′ UTR.

Double-mutation line pBA002a-P_CHR2_-LUC-FM-pri-miR159a-T1-DM-3′ UTR was introduced by PCR using pBA002a-P_CHR2_-LUC-FM-pri-miR159a-T1-3′ UTR as template. Then the fragments containing point mutations were swapped into pBA002a-P_CHR2_-LUC-FM-pri-miR159a-T1-3′ UTR using restriction enzyme digestion followed by ligation to yield pBA002a-P_CHR2_-LUC-FM-pri-miR159a-T1-DM-3′ UTR.

The pBA002a-DC-FM-3′ UTR was described previously [19]. The pBA002a-P_FT_-FT-FM-3′ UTR and its derivatives were constructed as follows: first, FT promoter was amplified with primers PFT For and PFT Rev (Additional file 2: Table S1), and fused with EcoRV-digested pBA002a-DC-FM by NEBuilder HiFi DNA Assembly Master Mix (NEB) to generate pBA002a-P_FT_-DC-FM. Meanwhile, FT coding sequence was amplified with primers FT For and FT Rev and transferred into pBA002a-P_FT_-DC-FM by LR reaction to yield pBA002a-P_FT_-FT-FM. Then, 3′ UTR, pri-miR159a-3′ UTR, and pri-miR159a-T1-3′ UTR fragments were amplified from plasmid P_CHR2_-LUC-FM-3′ UTR, pBA002a-P_CHR2_-LUC-FM-pri-miR159a-3′ UTR, and pBA002a-P_CHR2_-LUC-FM-pri-miR159a-T1-3′ UTR, respectively, and then fused with PacI-digested pBA002a-P_FT_-FT-FM by NEBuilder HiFi DNA Assembly Master Mix (NEB) to generate pBA002a-P_FT_-FT-FM-3′ UTR and its derivates.

### Selection of single copy of transgenes, and LUC assays

The LUC activity results mentioned above were obtained from a large population of transgenic plants. To minimize the potential impact of transgenic copy numbers and positional effects of transgene insertions, transgenic plants with a single copy of the transgene were further screened based on genetic segregation. Briefly, transgenic plants were propagated on Basta (glufosinate ammonium) plates, and the lines with a mortality rate of 25% or more were retained. In the T3 generation, the lines that all survived (0% mortality rate) on Basta plates were considered as single-copy lines.

For LUC activity assays, 6-day-old transgenic seedlings from T1, T2, and T3 generations were examined with an electron-multiplying charge-coupled device (CCD) camera and WinView 32 and LightField (LightField Version: 6.4.1.1709. link of the website: https://www.princetoninstruments.com/products/software-family/lightfield). LUC images in Figs. 1a, c, and 2c, and Additional file 1: S1a were captured via an older version of a CCD camera (Olympus DP70), whereas the others were done later on via an upgraded CCD camera system (Schneider Kreuznach). In each LUC assay, we always captured LUC images using the same CCD camera and with the same parameters. In addition, we compared the LUC intensity of different lines to their own control (CK, *P*_*CHR2*_*-LUC-3*′ *UTR*) to ensure consistency in each experiment. The relative LUC signal activity was quantified based on the luminescence intensity of the LUC signal.

### Northern blot analysis

Total RNA was extracted using TRIzol reagent (Sigma, T9424) from 6-day-old seedlings. Northern blot hybridization was performed as described previously [71]. The probes for detecting *LUC* transcript were PCR products that were amplified using primers detailed in Additional file 2: Table S1. The probes were then labeled by [α-32P]2′-deoxycytidine 5′-triphosphate(dCTP) (PerkinElmer) with Klenow fragment (3′ to 5′ exo-; NEB). Hybridization signals were detected with Typhoon FLA 7000 (GE Healthcare).

### Chromatin immunoprecipitation–polymerase chain reaction

The ChIP assay was performed as described previously [72]. Briefly, 10-day-old seedlings were harvested from MS medium and crosslinked to stabilize DNA–protein complexes. The samples were then fragmented, and DNA-Pol II complexes were immunoprecipitated using anti-Pol II antibody (Abcam, ab252854). The DNA was extracted and used for quantitative PCR to detect the abundance of Pol II in various regions of the *LUC* locus in each line. PCR primers are listed in Additional file 2: Table S1.

### Western blot analyses

Western blot analyses for Col-0; *P*_*FT*_*-FT-FM-3*′ *UTR*, Col-0; *P*_*FT*_*-FT-FM-pri-miR159a-3*′ *UTR*, and Col-0; *P*_*FT*_*-FT-FM-pri-miR159a-T1-3*′ *UTR* were performed as described previously [70]. The blots were detected with antibodies against Myc (Sigma-Aldrich, C3956) and actin (Sigma-Aldrich, A0480). Secondary antibodies were goat-developed anti-rabbit (GE Healthcare, NA934) and anti-mouse immunoglobulin G (GE Healthcare, NA931). Western blot membranes were developed with ECL+, and signals were detected with ChemiDoc XRS+ and captured with the Image Lab software (Bio-Rad) in accordance with the manufacturer’s instructions.

### RNA extraction, reverse transcription, and quantitative real-time PCR (qRT-PCR)

Total RNA was extracted using TRIzol reagent (Sigma, T9424) from plant samples. cDNA synthesis and qRT-PCR were performed as previously described [70]. Primers are listed in Additional file 2: Table S1.

### 3′ RACE to identify polyadenylation sites

A method modified from a 3′ RACE experiment was used to identify polyadenylation sites. In brief, 10 μg of total RNA was used to synthesize cDNAs using 3′ adapter primer (Additional file 2: Table S1). The 3′ end cDNA was then amplified for two rounds using two sets of primers (LUC-outer For and 3′ adapter Rev, LUC-inner For and 3′ adapter Rev) listed in Additional file 2: Table S1. The resulting PCR products were cloned into the pENRT vector, and the distinct clones were sequenced using either M13 forward or M13 reverse primers to obtain their corresponding sequences.

### In vivo mRNA decay assay

Ten-day-old seedlings were harvested from MS plates and transferred to incubation buffer (15 mM sucrose, 1 mM KCl, 1 mM PIPES pH 6.25, 1 mM sodium citrate) in a 12-well culture plate, where they were pre-soaked for 30 min. Transcription inhibitor Actinomycin D (final concentration, 50 μM, Sigma-Aldrich, A1410) was then added to each reaction and mixed well. The seedlings were subjected to a vacuum condition and swirled every 7.5 min during a 15-min incubation period. Samples were harvested at various time points, 0 h, 0.5 h, 1 h, 2 h, and 6 h.

### In vitro transcription, infiltration, and mRNA decay assay

In vitro transcription was performed as described [20]. RNA substrates were transcribed under the T7 promoter in vitro using PCR-amplified templates. The primers used for PCR are listed in Additional file 2: Table S1.

To determine the decay rate of the two in vitro transcripts (3′ end regions of *P*_*CHR2*_*-LUC-3*′ *UTR* and *P*_*CHR2*_*-LUC-pri-miR159a-T1-3*′ *UTR*), 10-day-old Col-0, *xrn4*, and *sov* seedlings grown on MS medium were harvested and transferred to incubation buffer (15 mM sucrose, 1 mM KCl, 1 mM PIPES pH 6.25, 1 mM sodium citrate) in a 12-well culture plate. In vitro transcripts (final concentration, 1 μg/mL) were then added separately to the reactions. Another in vitro transcribed *LUC* fragment was included as a reference transcript to assess infiltration efficiency. The seedlings were then incubated for 15 min under a vacuum condition and swirled every 7.5 min. Samples were then collected at various time points, 0 h, 0.5 h, 1 h, 2 h, and 6 h.

To verify the infiltrated efficiency of in vitro transcripts, equal amounts of in vitro transcripts were either infiltrated (under vacuum conditions for 15 min) or un-infiltrated (soaked for 15 min) into 10-day-old Col-0 seedlings. After treatment, plants were then washed three times in ddH_2_O to remove the potential remaining transcripts stuck on the surface of the plants. The treated seedlings, the in vitro transcripts leftover in the incubation solution and subsequent washing ddH_2_O were collected for qRT-PCR analysis.

### In vivo DMS modification

In vivo DMS modification was performed as described [19]. Ten-day-old seedlings grown on MS plates were harvested and covered in 15 mL of 1 X DMS reaction buffer (40 mM HEPES pH 7.5, 100 mM KCl, 0.5 mM MgCl_2_) in a 50-mL Corning tube. To the DMS-treated or DMS-nontreated samples, 150 μL of DMS (final concentration of 1%, Sigma, D186309) or deionized water was added separately and mixed thoroughly. Samples were then incubated for 15 min under a vacuum condition and swirled every 7.5 min. To quench the reaction, 5 mL β-mercaptoethanol was added to a final concentration of 20% and incubated under vacuum for 5 min.

### In vivo and in vitro 3′ end target-specific DMS-MaPseq

The 3′ end target-specific DMS-MaPseq protocol was adapted from a previous study [24]. For in vivo RNA structure probing, briefly, 10-day-old seedlings were treated with 1% DMS (DMS-treated) or deionized water (DMS-untreated) for 15 min under vacuum. Total RNA was extracted using TRIzol reagent (Sigma). Polyadenylated RNAs were purified from 10 μg of TURBO DNase-treated total RNA using poly(T)25 Dynabeads (Life Technology) with elution in 11 μL DEPC-H_2_O. For in vitro RNA structure probing, polyadenylated RNAs were enriched from 10 μg of TURBO DNase-treated total RNA using poly(T)25 Dynabeads (Life Technology). Half of the RNA was then refolded in refolding buffer (10 mM Tris-HCl pH 8.0, 20 mM NaCl, 1 mM EDTA) by heating at 95 °C for 2 min and then slowly cooling down. The refolded RNA was treated with 0.3% DMS for 3 min in an Eppendorf tube. After treatment, the reaction was immediately quenched by 0.5 M DTT, followed by phenol–chloroform extraction and precipitation.

The probed RNA was then reverse transcribed using 1 μL 50 μM 3′ adapter primer (Additional file 2: Table S1), 1 μL 10 mM dNTPs, 4 μL 5X First-Strand buffer (250 mM Tris-HCl pH 8.3, 375 mM KCl, 15 mM MgCl_2_), 1 μL 0.1 M DTT, 1 μL SUPERase-In (Life Technology), and 1 μL TGIRT-III (Ingex, TGIRT50). The reaction was incubated at 42 °C for 30 min followed by 60 °C for 1.5 h. The RNA was hydrolyzed by adding 2.3 μL 1 M NaOH and heating at 98 °C for 15 min. After neutralization with 2.5 M HCl, the mixture was cleaned using RNAClean XP beads (Beckman Coulter) and resuspended in deionized water. The resulting cDNA was then amplified for two rounds using two sets of primers (LUC-outer For and 3′ adapter Rev, LUC-inner For and 3′ adapter Rev) in Additional file 2: Table S1. The PCR products were purified using AMPure XP beads (Beckman Coulter) and normalized before library construction.

Purified PCR products were then sonicated into fragments of 150–300 bp, followed by end repair, adenylation, and adapter ligation using Illumina adapters, mainly following a previously published protocol [73]. The resulting fragments were barcoded through adapter ligation and the purified barcoded libraries were enriched by 15 cycles of PCR using KOD Hot Start DNA polymerase. The PCR products were cleaned using AMPure XP beads (Beckman Coulter) and submitted for Illumina sequencing.

### In vivo and in vitro 3′ end target-specific DMS-MaPseq data analysis

To ensure the reliability of the sequencing data, we obtained an average of 10 million paired-end 150 bp reads for each sample of 3′ end target-specific DMS-MaPseq using Illumina Novaseq 6000. The raw reads were subjected to quality control by trimming the Illumina universal adapter and low-quality ends with a quality score < 25 using cutadapt [74]. To further improve the sequence quality, “Fastq_quality_filter” from the “Fastx-toolkit” [75] was used to filter sequences with low quality with the parameters “-q 25 -p 80”, indicating that 80% of the nucleotides had a base call accuracy of more than 99.7%. TopHat2 (v2.1.2) [76] was utilized to map the reads against specific plasmid sequences with the parameters “-N 15 –read-gap-length 10 –read-edit-dist 15 –max-insertion-length 5 –max-deletion-length 5 -g 3” and only uniquely mapped reads were retained for further analysis.

To call mismatches, we employed a homemade python script called “CountMismatch2Bed.py” (https://github.com/changhaoli/TAMU_02RSS). This script is user-friendly and outputs a bed file containing the location and mismatch count information when given a bam file. To obtain the coverage for each nucleotide, we used BEDTools (v2.29.2) [77] subfunction “genomecov” with the parameters “-d -split”. Raw DMS reactivity was calculated as the ratio between the mismatch count and the coverage for each nucleotide. Normalized DMS reactivity was obtained by dividing the raw DMS reactivity by the median of the top 5% raw DMS reactivities. Ratios greater than 1.0 were set to 1.0. We then calculated the average DMS activities of A/C/G/U and plotted the histograms for each sample using “ggpubr” [78]. Plotly R (https://plotly.com/r/) was used to create 3D plots based on DMS reactivities for three biological replicates with different vectors, and we calculated the Pearson correlation (*R* value) for each pair.

Because of the high reproducibility of our 3′ target-specific DMS-MaPseq, we merged the three biological replicates. Sliding-window method [24] was used to calculate the Gini index for each sample based on a previous method with some modifications. Specifically, each target sequence was divided into 50-nt windows with a 25-nt step, and the Gini index was calculated for each window. Only windows with an average of more than 20 mismatches were used for further analysis. Boxplots for each treatment were created using “ggpubr”.

### DIM-2P-seq

DIM-2P-seq was slightly modified from [2]. Three-week-old plants were treated with 1% DMS (DMS-treated) or deionized water (DMS-untreated) for 15 min under vacuum. Total RNA was extracted using TRIzol reagent (Sigma). Ten micrograms RNA was used to perform DIM-2P-seq. Briefly, polyadenylated RNAs were selected by poly(T)25 Dynabeads (Life Technology) per the manufacturer manual. The selective RNAs were reverse transcribed using 1 μL RT primer (Additional file 2: Table S1), 1 μL 10 mM dNTPs, 4 μL 5X First-Stand buffer, 1 μL 0.1 M DTT, 1 μL SUPERase-In (Life Technology), and 1 μL TGIRT-III (Ingex, TGIRT50). The reaction was incubated at 42 °C for 30 min followed by 60 °C for 1.5 h. Then the RNA template was hydrolyzed by adding 2.3 μL 1 M NaOH and incubated at 98 °C for 15 min. cDNAs were resolved on 6% TBE-Urea PAGE Gel, and those with length above 150 nt were extracted and then circularized in a reaction containing 15 μL cDNA, 2 μL 10 X CirLigase buffer, 1 μL 1 mM ATP, 1 μL 50 mM MnCl_2_, and 1 μL CirLigase (Biosearch Technologies, CL4115K), at 60 °C for 4 h and followed by 80 °C for 10 min. The circularized cDNA was used for 15 cycles of PCR amplification in which barcoded Illumina adapters were added. The PCR products between 300 and 650 bp were extracted from agarose gels and submitted for Illumina sequencing (PE150, Illumina Novaseq 6000).

### DIM-2P-seq data analysis

The raw reads of DIM-2P-seq underwent the same sequence quality control process as 3′ end target-specific DMS-MaPseq. This involved trimming adapters and filtering low-quality reads, followed by mapping high-quality reads to the *Arabidopsis* genome Araport11 [44] using TopHat2 (v2.1.2) with the same parameters as 3′ end target-specific DMS-MaPseq. Only uniquely mapped reads were retained for subsequent analyses. The sample clustering was analyzed by treating DIM-2P-seq as RNA-seq, using “featureCounts” [79] to obtain read counts, and the DESeq2 RNA-seq workflow [80] to draw the sample clustering heatmap based on normalized read counts. The “deepTools” software [81] assessed the distribution of the reads for DIM-2P-seq and RNA-seq. The RNA-seq data used in this study was previously published [34].

Average DMS reactivities for the first, second, and third nucleotides for all codons within the 3′ end region were calculated based on the meta-gene plot data. For the 3′ UTR part, the first, second, and third nucleotides were defined as the nucleotides being just downstream stop codon. These average DMS reactivities for the nucleotide positions were used to draw the bar chart.

The same sliding-window method as for 3′ end target-specific DMS-MaPseq was used to calculate the Gini index for DMS reactivities from DIM-2P-seq. Only windows with an average of more than 20 mismatches were retained for further analysis. For each 3′ UTR, the average Gini index was calculated based on its own windows. The genes with the top 10% of the high 3′ UTR Gini index and the bottom 10% of the low 3′ UTR Gini index were defined as the high-Gini and low-Gini genes, respectively. The expression levels for these genes were obtained from our previously published paper [34]. A cumulative curve and boxplot were drawn based on the Gini index and expression levels (RPKM) for the low-Gini and high-Gini genes. Metascape [82] was used to perform GO enrichment analysis for the low-Gini and high-Gini genes. For human DMS-MaPseq data, the same processing procedure was employed to obtain the low-Gini and high-Gini genes. DMS-MaPseq was used as RNA-seq to obtain gene expression. For rice DMS-seq, the pipeline from “RNA Framework” [83] was applied to obtain DMS reactivities, and gene expression was calculated based on DMS-seq. A cumulative curve and boxplot were drawn using the same method as described above. Scatter plots between 3′ UTR Gini index and RNA expression levels (RPKM) were drawn via “ggscatter” of “ggpubr” [78].

To investigate the potential correlation between Gini index and RNA half-life, we obtained two publicly available RNA decay datasets (GSE86361 and GSE136713) [46, 47] to quantify RNA transcripts with long half-life (the top 10%) and short half-life (the bottom 10%). Subsequently, we generated cumulative curves to compare the distribution of Gini index between these two groups of genes. We also compare Gini index for the transcripts with longer half-life in *sov* (the top 10% of log_2_(*sov*/WT)) and those with shorter half-life in *sov* (the bottom 10% of log_2_(*sov*/WT)) [47]. For these analyses, only transcripts with an average Gini index of more than 0.4 were studied.

### Confounding factors analysis

GC content was calculated by SeqKit [84] with 3′ UTR sequences as input. Genes with GC content ≥ 32.55% (median of GC content) were defined as the high-GC genes, while others were the low-GC genes. Boxplot was used to compare the difference of GC content between the high-Gini and low-Gini genes.

The data of poly(A) tail length was acquired from previous publication [40]. Genes with poly(A) tail length ≥ 80 nt (median of poly(A) tail length) were defined as the long poly(A) tail genes, while others were the short poly(A) tail genes. Boxplot was employed to compare the poly(A) tail length between the high-Gini and low-Gini genes.

The lengths of 3′ UTRs from the low-Gini and high-Gini genes were calculated based on *Arabidopsis* Araport11 annotation. Boxplot was used to compare the difference of 3′ UTRs length between these two groups.

A previous study showed RG4 in 3′ end of transcripts enhanced mRNA stability [42], we wondered whether our high-Gini and low-Gini genes contain RG4 or not. The software “quadparser” [43] was used to predict all RG4 sites with a total number of 2722. Overlaps were performed between all RG4 sites and 3′ UTRs of the high-Gini and low-Gini genes.

Information of miRNA target sites was retrieved from TarDB database [85]. Pie chart was used to show the percentage of miRNA target sites for the high-Gini and low-Gini genes.

### RNA secondary structures modeling

For 3′ end target-specific DMS-MaPseq, we first normalized the DMS mismatch ratio by DMS-untreated *P*_*CHR2*_*-LUC-3*′ *UTR* and then further normalized by the median of the greatest 5% of mutation rates (The nucleotides with reactivities larger than 1.5 times the inter-quartile range are identified as outliers [86]). For DIM-2P-seq, we normalized the DMS mismatch ratio by the median of the greatest 5% of mutation rates. Normalized rates greater than or equal to 1.0 were set to 1.0. We utilized RNAstructure command line program (Mathews lab) [23] to model their RSS of 3′ UTR regions (from the region after the stop codon to the poly(A) site) based on the DMS mismatch ratio at each nucleotide.

All the RNA secondary structures in this study were predicted by “RNAstructure” software [23], and the RNA structure with the lowest thermodynamic energy was shown. The base-pairing probabilities were calculated by the command “ProbablePair” also from “RNAstructure”.

### Statistical analysis

For 3′ end target-specific DMS-MaPseq and DIM-2P-seq, the data were analyzed by performing a Wilcoxon rank sum test using RStudio (Version 3.6). The threshold for determining significant differences was *P* < 0.05.

For ChIP-qPCR and qRT-PCR, the data were presented as means of at least three biological replicates ± SE. For Additional file 1: Fig. S1b, the relative abundance of Pol II in different loci was first normalized to input, and then to that of *UBQ10*. For Fig. 3, the relative expression of tested genes was normalized to that of *18S rRNA* (Fig. 3a, c) and in vitro transcribed *LUC* segments (Fig. 3b). For Fig. 6b, the relative *FT* expression was normalized to that of *UBQ10*. For Additional file 1: Fig. S3d, relative in vitro transcripts amount in different treatments (infiltration and un-infiltration) in Col-0 was normalized to that of *UBQ10*. In vitro transcripts leftover in the incubation solution and subsequent washing solution were normalized to the amount in the initial incubation solution. Unpaired two-tailed Student’s *t* test was performed to calculate the *P* value. The cutoff for significance was 0.05. ns, no significance; *P < 0.05; **P < 0.01; ***P < 0.001.

For quantification of luminescence, each point represented an individual line with a mean of 10–12 individual plants. Eight to sixteen individual lines were utilized for each transgenic line. Unpaired two-tailed Student’s *t* test was performed to calculate the *P* value. The cutoff for significance was 0.05. ns, no significance; *P < 0.05; **P < 0.01; ***P < 0.001.

### Supplementary Information

**Additional file 1: Fig. S1.** *LUC* expression is unaffected by Microprocessor function, RNA Pol II transcription, or miPEPs effect. **Fig. S2.** High-quality and reproducibility of 3’ end target-specific DMS-MaPseq datasets. **Fig. S3.** Transgenic plants with truncation segments of poorly structured 3’ UTRs of pri-miR159a have high *LUC* expression. **Fig. S4.** Quality control analysis of DIM-2P-seq datasets. **Fig. S5.** Negative relationship between RSS of 3’ UTRs and transcript expression level is not confounded by selected factors. **Fig. S6.** RSS of 3’ UTRs is inversely correlated with transcripts half-life.

**Additional file 2: Table S1.** Summary of primer sequences.

**Additional file 3.** Vector sequences of transgenic lines employed in 3’ end target-specific DMS-MaPseq.

**Additional file 4.** Uncropped images for the blots in Figs. 1 and 6.

**Additional file 5.** Review history.

## Data Availability

The raw sequencing data of in vivo and in vitro 3′ end target-specific DMS-MaPseq and DIM-2P-seq from this study have been deposited in the National Center for Biotechnology Information Sequence Read Archive (https://www.ncbi.nlm.nih.gov/bioproject/PRJNA1049869) under SRA accession PRJNA1049869 [87]. The published RNAseq data of *Arabidopsis* were downloaded from NCBI SRA database under PRJNA613247 [34]. The published DMS-seq data of rice and DMS-MaPseq data of human were downloaded from NCBI SRA database under PRJNA401523 [35] and PRJNA396539 [36], respectively. The published RNA decay RNAseq results for *Arabidopsis* were obtained from published papers [46, 47].
