# Supplementary material for: Structured 3′ UTRs destabilize mRNAs in plants
Source: Genome Biol. 2024 Feb 22;25:54. doi: 10.1186/s13059-024-03186-x (PMC10885604; doi:10.1186/s13059-024-03186-x)
Supplement: Supplementary file 1 — Additional file 1: Fig. S1. LUC expression is unaffected by Microprocessor function, RNA Pol II transcription, or miPEPs effect. Fig. S2. High-quality and reproducibility of 3’ end target-specific DMS-MaPseq datasets. Fig. S3. Transgenic plants with truncation segments of poorly structured 3’ UTRs of pri-miR159a have high LUC expression. Fig. S4. Quality control analysis of DIM-2P-seq datasets. Fig. S5. Negative relationship between RSS of 3’ UTRs and transcript expression level is not confounded by selected factors. Fig. S6. RSS of 3’ UTRs is inversely correlated with transcripts half-life. [file 13059_2024_3186_MOESM1_ESM.docx]

**Additional file 1: Supplementary figures**

**Title: Structured 3’ UTRs destabilize mRNAs in plants**

**Authors:** Tianru Zhang^1,2,9^, Changhao Li^1,9^, Jiaying Zhu^1,9*^, Yanjun Li^3^, Zhiye Wang^4^, Chun-Yip Tong^1^, Yu Xi^5^, Yi Han^6^, Hisashi Koiwa^7^, Xu Peng^5^, and Xiuren Zhang^1,2,8*^

**Affiliations:**

^1^Department of Biochemistry and Biophysics, Texas A&M University, College Station, TX 77843, USA.

^2^Molecular and Environmental Plant Sciences, Texas A&M University, College Station, TX 77843, USA.

^3^State Key Laboratory for Managing Biotic and Chemical Threats to the Quality and Safety of Agro-products, Institute of Plant Virology, Ningbo University, Ningbo 315211, China.

^4^State Key Laboratory of Plant Physiology and Biochemistry, College of Life Sciences, Zhejiang University, Hangzhou 310058, China.

^5^Department of Medical Physiology, College of Medicine, Texas A&M University, Bryan, TX 77807, USA.

^6^National Engineering Laboratory of Crop Stress Resistence Breeding, School of Life Sciences, Anhui Agricultural University, 230036 Hefei, China.

^7^Department of Horticultural Sciences, Texas A&M University, College Station, TX77843, USA.

^8^Department of Biology, Texas A&M University, College Station, TX 77843, USA.

^9^These authors contributed equally to this work.

*To whom correspondence may be addressed. Email: [zhujiaying@tamu.edu](mailto:zhujiaying@tamu.edu) or [xiuren.zhang@tamu.edu](mailto:xiuren.zhang@tamu.edu)

**Supplemental Figures and Figure Legends**

**Additional file 1: Fig. S1 *LUC* expression is unaffected by Microprocessor function, RNA Pol II transcription, or miPEPs effect.**

**a** LUC signals of T2 three-week-old transgenic plants (left panel). Quantification of luminescence results from different transgene lines (right panel). *dcl1-9* and *se-3* homozygotes (-/-) displayed small statues and were circled in red. Scale bar, 0.5 cm. Statistical test was performed among different transgenic lines. ns, no significance; unpaired two-tailed Student’s *t*-test.

**b** 3’ RACE experiment identified poly(A) sites of different transgenic lines (*P_CHR2_-LUC-3’ UTR*, *P_CHR2_-LUC-pri-miR159a-3’ UTR*, *P_CHR2_-LUC-pri-miR159a-T1-3’ UTR*, *P_CHR2_-LUC-pri-miR159a-T1-1-3’ UTR*, and *P_CHR2_-LUC-pri-miR164a-3’ UTR*). (Left panel) Gel images showed PCR product length. (Right panel) Red lightning arrows pointed to poly(A) sites of each line in schematics.

**c** Double missense mutations of start codons for two hypothetic miPEPs in pri-miR159a-T1 did not alter the patterns of luminescence compared with *P_CHR2_-LUC-pri-miR159a-T1-3’ UTR*. The locations of ORFs predicted to decode two miPEPs were shown in schematics (upper panel). Quantification of luminescence results from different transgene lines (lower panel). Exposure time of LUC signals from CCD camera was 20 S. DM, double mutations. Statistical test was performed between different transgenic lines and *P_CHR2_-LUC-3’ UTR*. ns, no significance; **P* < 0.05; ***P* < 0.01; ****P* < 0.001; unpaired two-tailed Student’s *t*-test.

**d** ChIP–qPCR assay did not reveal significant change of RNA polymerase II occupancy in *CHR2* promoter (*P_CHR2_*) and *LUC* locus in different transgenic lines (*P_CHR2_-LUC-3’ UTR*, *P_CHR2_-LUC-pri-miR159a-3’ UTR*, and *P_CHR2_-LUC-pri-miR159a-T1-3’ UTR*). Regions selected for ChIP-qPCR analysis were marked in the graphs (left panel). ChIP signal was first normalized to input, and then to that of *UBQ10*. *UBQ10* serves as a positive control. ChIP without the antibody (no AB) served as negative controls. The data were presented as means ± SE (n = 3) biologically independent replicates.

In (**a**) and (**c**), whiskers represent the minimum and maximum values whereas horizontal lines in the boxplots display the 75^th^, 50^th^, and 25^th^ percentiles, respectively.

**Additional file 1: Fig. S2 High-quality and reproducibility of 3’ end target-specific DMS-MaPseq datasets.**

**a** DMS reactivity correlations among three biological replicates for different transgenic lines (*P_CHR2_-LUC-3’ UTR*, *P_CHR2_-LUC-pri-miR159a-3’ UTR*, *P_CHR2_-LUC-pri-miR159a-T1-3’ UTR*, and *P_CHR2_-LUC-pri-miR159a-T1-1-3’ UTR*, and *P_CHR2_-LUC-pri-miR164a-3’ UTR*). *R* value is the Pearson’s correlation coefficient value.

**b** Average normalized DMS reactivities of nucleotides from different transgene lines in (A) vs the reference transcripts without DMS treatment. One DMS-untreated (non) and three DMS-treated biological replicates (R1, R2, and R3) were shown.

**c** The secondary structure of nucleotides 121-186 of *CAB1* (*At1g29930*) mRNA was determined using DMS reactivities from this study. DMS reactivities from gel-based RNA structure probing method were extracted [1]. Pearson correlation coefficient (PCC) of DMS reactivity was calculated between this study and a published paper [1]. DMS reactivity ≥ 0.8, 0.6, 0.3~0.6, and 0~0.3 were marked in red, pink, orange, and green, respectively with U/G bases in gray.

**Additional file 1: Fig. S3 Transgenic plants with truncation segments of poorly structured 3’ UTRs of pri-miR159a have high *LUC* expression.**

**a** 3’ RACE experiment identified poly(A) sites of *LUC* transcripts from different transgenic lines. The poly(A) sites of *P_CHR2_-LUC-pri-miR159a-T2-3’ UTR*, *P_CHR2_-LUC-stem-loop-3’ UTR*, and *P_CHR2_-LUC-pri-miR159a-T2-2-3’ UTR* were sequenced by Sanger sequencing, and *P_CHR2_-LUC-pri-miR159a-T2-1-3’ UTR* was identified from high throughput sequencing.

**b** Schematic constructs and the predicted secondary structure models of the 3’ UTR for different transgenic lines (*P_CHR2_-LUC-3’ UTR*, *P_CHR2_-LUC-pri-miR159a-T2-3’ UTR*, *P_CHR2_-LUC-stem-loop-3’ UTR*, *P_CHR2_-LUC-pri-miR159a-T2-1-3’ UTR*, and *P_CHR2_-LUC-pri-miR159a-T2-2-3’ UTR*). The DMS signal of A and C residues were color-coded and U/G bases were marked in gray. Exposure time of LUC signals from CCD camera was 30 S. For different truncated segments of pri-miR159a, the red lines represented the retained regions of pri-miR159a, while the gray regions were removed in the constructs.

**c** Predicted base-pairing probabilities of different transgenic lines in (**a**) via RNAstructure [2]. *P* values by Wilcoxon test. Horizontal lines in the boxplots display the 75^th^, 50^th^, and 25^th^ percentiles, respectively. The upper fence is 75^th^ percentile +1.5 * interquartile range. The lower fence is 25^th^ percentile -1.5 * interquartile range. Dots represent the outliers.

**d** qRT-PCR assays validated the delivery efficiency of *in vitro* transcripts into plant tissues via vacuum infiltration. (Left panel) A significantly higher level of *in vitro* transcripts was detected inside the infiltrated samples vs the un-infiltrated samples. (Right panel) A significantly reduced amount of leftover *in vitro* transcripts was detected in the incubation solution and subsequent washing solution for the infiltrated samples vs the un-infiltrated ones. Equal amounts of *in vitro* transcripts were either infiltrated (under vacuum conditions for 15 minutes) or left un-infiltrated (soaked for 15 minutes) in 10-day-old Col-0 seedlings. After the treatment, plants were then washed three times in ddH_2_O before sampling. Relative *in vitro* transcripts amount in different treatments in Col-0 was normalized to that of *UBQ10*. *In vitro* transcripts leftover in the incubation solution, in 1^st^ washing ddH_2_O, and in 3^rd^ washing ddH_2_O were normalized to the amount in the initial incubation solution. The data were presented as means ± SE (n = 3) biologically independent replicates.

**Additional file 1: Fig. S4 Quality control analysis of DIM-2P-seq datasets.**

**a** Heatmap of sample-to-sample distances among one DMS-untreated (Untreated) and three DMS-treated samples (Rep1, Rep2, and Rep3). Noted: the three DMS-treated samples were clustered together, and distinct from the untreated sample.

**b** Average metagene profiles for DIM-2P-seq (left part) and RNA-seq (right part, PRJNA613247) show reads enrichment patterns on 5’ UTR, CDS, and 3’ UTR.

**c** High reproducibility of DMS reactivity correlation among three biological replicates for DIM-2P-seq with Pearson’s correlation coefficient *R* of more than 0.98.

**d** Average DMS reactivities of A, C, G, and U for DIM-2P-seq indicate a high signal-to-noise ratio of A/C (average DMS reactivity of more than 0.04) versus G/U (average DMS reactivity less than 0.01) in three DMS-treated replicates.

**e** The secondary structures of U1 snRNA and nucleotides 121-186 of *CAB1* mRNA (At1g29930) were determined using DMS reactivities. DMS reactivities from gel-based RNA structure probing method were extracted [1]. PCC was calculated between our data and published data [1]. DMS reactivity ≥ 0.8, 0.6, 0.3~0.6, and 0~0.3 were marked in red, pink, orange, and green, respectively with U/G bases in gray.

**f** Average DMS reactivities of the first, second, and third nucleotide in codons for CDS and 3’ UTR to show the periodicity difference. *P* values by Wilcoxon test.

**Additional file 1: Fig. S5 Negative relationship between RSS of 3’ UTRs and transcript expression level is not confounded by selected factors.**

**a-c** Scatter plots of correlation analysis between 3’ UTR RSS (average Gini) and RNA expression level (log_10_(RPKM)) in *Arabidopsis* (**a**), rice (**b**), and human (**c**). Be noted that RSS of 3’ UTRs was decoded through a TGIRT-mutational profiling method in *Arabidopsis* and human but through a regular reverse transcription-stop method in rice. *R*, Pearson’s correlation coefficient. *P* was calculated by Pearson’s correlation analysis.

**d** (Left panel) Comparison of 3’ UTR GC content (%) between the high-Gini vs the low-Gini genes. (Middle panel) Genes were categorized into high and low GC content, with half of the genes having GC content ≥ 32.55% and half having < 32.55%. Boxplots were used to compare the difference of GC content between the high-Gini genes and the low-Gini genes. (Right panel) Comparison of gene expression level (RPKM) between the high-Gini and the low-Gini genes in two different GC content categories. *P* values by Wilcoxon test.

**e** (Left panel) Comparison of poly(A) tail length between the high-Gini vs the low-Gini genes. (Middle panel) Genes were classified into the long and short poly(A) tail length, with half of the genes having poly(A) tail length ≥ 80 and half having < 80 nt. Boxplots were used to compare the difference in poly(A) tail lengths between the high-Gini genes and the low-Gini genes. (Right panel) Comparison of gene expression level (RPKM) between the high-Gini and the low-Gini genes in two different poly(A) tail length categories. *P* values by Wilcoxon test. Poly(A) tail length data was acquired from previous publication [3].

**f** 3’ UTR length between the high-Gini vs the low-Gini genes. *P* values by Wilcoxon test.

**g** Overlaps between RG4 sites with the high-Gini or the low-Gini genes. RG4: RNA G-quadruplex.

**h** Percentages of miRNA target sites in the high-Gini and the low-Gini 3’ UTRs.

In (**d**-**f**), horizontal lines in the boxplots display the 75^th^, 50^th^, and 25^th^ percentiles, respectively. The upper fence is 75^th^ percentile +1.5 * interquartile range. The lower fence is 25^th^ percentile -1.5 * interquartile range. Dots represent the outliers.

**Additional file 1: Fig. S6 RSS of 3’ UTRs is inversely correlated with transcripts half-life.**

**a** Comparison of Gini indexes between short and long half-life genes in *Arabidopsis* (RNA decay data, GSE136713). *P* value by Kolmogorov-Smirnov test.

**b-c** Scatter plots of correlation analysis between 3’ UTRs RSS (average Gini) and RNA half-life (log_10_(half-life min)) of published data GSE86361 (**b**) and GSE136713 (**c**). *R*, Pearson’s correlation coefficient. *P* was calculated by Pearson’s correlation analysis.

**d** Scatter plot of correlation analysis between 3’ UTRs RSS (average Gini) and RNA half-lives fold change (*sov*/WT) in *Arabidopsis* (RNA decay data, GSE86361). *R*, Pearson’s correlation coefficient. *P* was calculated by Pearson’s correlation analysis.

**e** Motifs enriched by MEME for the high-Gini and the low-Gini 3’ UTRs.

**Reference**

1. Ding Y, Tang Y, Kwok CK, Zhang Y, Bevilacqua PC, Assmann SM. In vivo genome-wide profiling of RNA secondary structure reveals novel regulatory features. Nature. 2014,505:696-700.

2. Reuter JS, Mathews DH. RNAstructure: software for RNA secondary structure prediction and analysis. BMC Bioinformatics. 2010,11:129.

3. Jia J, Lu W, Liu B, Fang H, Yu Y, Mo W, Zhang H, Jin X, Shu Y, Long Y, et al. An atlas of plant full-length RNA reveals tissue-specific and monocots-dicots conserved regulation of poly(A) tail length. Nat Plants. 2022,8:1118-1126.
