# Supplementary material for: Structured 3′ UTRs destabilize mRNAs in plants
Source: Genome Biol. 2024 Feb 22;25:54. doi: 10.1186/s13059-024-03186-x (PMC10885604; doi:10.1186/s13059-024-03186-x)
Supplement: Supplementary file 4 — Additional file 4. Uncropped images for the blots in Figs. 1 and 6. [file 13059_2024_3186_MOESM4_ESM.docx]

Additional file 4: Uncropped images for the blots in Figure 1 and Figure 6.

Uncropped western blotting and gel for Fig. 1

Uncropped western blotting and gel for Fig. 6
