# Supplementary material for: Structured 3′ UTRs destabilize mRNAs in plants
Source: Genome Biol. 2024 Feb 22;25:54. doi: 10.1186/s13059-024-03186-x (PMC10885604; doi:10.1186/s13059-024-03186-x)
Supplement: Supplementary file 5 — Additional file 5. Review history. [file 13059_2024_3186_MOESM5_ESM.docx]

Review History

**First round of review**

**Reviewer 1**

**Are you able to assess all statistics in the manuscript, including the appropriateness of statistical tests used?** There are no statistics in the manuscript.

**Comments to author:**

The manuscript elucidates an inverse correlation between the 3' UTR RNA secondary structure and transcript accumulation in Arabidopsis. The authors incidentally discovered that the pri-miR159a embedded in the 3' UTR of CHR2 can lead to the accumulation of the harbored transcript. They further showed that poorly structured 3' UTR enhanced mRNA stability, resulting in an increased accumulation of transcripts in vivo. Whereas the highly structured 3' UTR tend to associate lower expression levels and a shorter half-life compared to their counterparts with poorly structured 3' UTR. Additionally, the authors engineered 3' UTR with different structures into an inherent FT gene and manipulated the flowering time in Arabidopsis. This work demonstrated the regulation of 3' UTR structure on gene expression level and offers a new strategy to modify plant traits in agricultural production and mRNA stability by engineering the 3' UTRs RSS in biotechnology. Nevertheless, there are a few questions I think the author need to address to improve the manuscript.

1. Figure 1a. The author should use the same CCD camera to get the same background for PCHR2-LUC-pri-miR164a-3'UTR and its control as the others. All images should be taken with the same CCD camera and ensure the consistency in both the camera settings and the imaging conditions. Figure 2c has a similar issue.

2. Line285. How did the author demonstrate the success of the infiltration of IVT RNA? I did not found any evidence in the manuscript. The author used in vitro transcribed LUC fragment as a reference transcript to assess infiltration efficiency. However, they did not provide evidence of the expression of LUC protein. They should provide a figure of LUC signals photographed under CCD camera.

3. Figure 3. How to explain the accumulation of the transcripts of pri-miR159a-T1-3'UTR after 1h infiltration in 3c. As the RNA is transcribed in vitro, its abundance cannot increase after infiltration. In addition, in the right panel of 3a, the error bar of pri-miR159a-T1-3'UTR at 0 h is too high.

4. L329-L344. It is hard to understand the higher expression of genes with poorly structured 3' UTR are involved in stress-related pathways. Since the gene expression data is obtained from Col-0 plants, other than stressed plants, the genes involved in stress-related pathway should not be induced and therefore should not display a high expression level.

5. Figure 3b. It is hard to distinguish the label of three groups lines, which is not consistent with the line chart, especially for the Col-0 (solid line) and sov (half dashed line).

6. In Method section, almost all the plasmids contained a "FM" element which is not appear in the main text. What did it indicate?

**Reviewer 2**

**Are you able to assess all statistics in the manuscript, including the appropriateness of statistical tests used?** Yes, and I have assessed the statistics in my report.

**Comments to author:**

**Summary:**

The study investigates the impact of RNA secondary structure (RSS) within 3' untranslated regions (3' UTRs) on mRNA stability and gene expression in Arabidopsis. It reveals that highly structured 3' UTRs lead to mRNA destabilization, while poorly structured 3' UTRs enhance mRNA accumulation. The research also indicates that this pattern extends from plants (Arabidopsis and rice) to mammals (human). The study suggests that manipulating 3' UTR RSS could be a potential strategy to modify gene expression and plant traits, offering implications for both agricultural production and biotechnology.

Overall Evaluation:

The presented study challenges the conventional understanding of the roles of RNA secondary structure (RSS) within 3' UTRs and offers insights into their influence on mRNA stability and gene expression. However, several aspects of the study should be further addressed before considering it for publication.

Detailed Comments:

1. Genomic Analysis and Confounding Factors:

The genomic analysis (Figures 4 and 5) constitutes only a smaller proportion of this study. The genomic analysis lacks consideration of potential confounding factors, which could impact the reported correlations. Controlling for such factors would strengthen the study's conclusions and improve the validity of the presented associations. Transcript properties are often associated with each other among genes to some degree, but many disappear when confounding factors are controlled for.

2. Data Presentation and Interpretation:

The classification of genes into categories in Figure 4c, e, f (and Figure 5a, b) appears arbitrary, making it difficult to evaluate the proportion of variation in mRNA levels that can be attributed to mRNA secondary structure. Encouraging the inclusion of scatter plots and correlation analysis would enhance the clarity and robustness of the presented data. In figure 5a, it is also not clear why ks test was used only for part of data.

3. Presentation Structure and Focus:

The study starts with an unexpected observation regarding pri-miR159a, which may confuse readers about the study's broader implications and its relevance to RNA secondary structure (RSS) within 3' UTRs in other sequence contexts. Providing a more coherent context and clearly establishing the relevance of the observed phenomenon would improve the study's overall structure.

4. Suggested Additional Analysis:

Related to the previous comment, considering the controversies in the field, the incorporation of Massively Parallel Reporter Assays could provide a robust validation of the findings and help resolve uncertainties related to the role of RNA secondary structure (RSS) in mRNA stability and gene expression.

5. 3'-UTR Terminology:

There is a misunderstanding in the terminology used for 3' UTRs throughout the manuscript. The study refers to the "end" of 3' UTRs as the point of transcription termination, whereas it should be clarified that it is the site of polyadenylation.

In conclusion, the study presents intriguing insights into the relationship between RNA secondary structure (RSS) within 3' UTRs and mRNA stability across various organisms. However, addressing the outlined concerns and refining the presentation will be crucial in enhancing the validity and impact of the findings.

**Reviewer 3**

**Are you able to assess all statistics in the manuscript, including the appropriateness of statistical tests used?** No, I do not feel adequately qualified to assess the statistics.

**Comments to author:**

The manuscript titled "DMS-MaPseq Implicates Structured 3' UTRs in mRNA Destabilization in Plants," authored by Zhang and colleagues, reveals that nuclear transcripts with weak secondary structures in their 3' UTRs tend to exhibit greater stability compared to those with highly structured 3' UTRs, thereby promoting gene expression.

Zhang et al. present compelling experiments, including generating transgenic plants with modified 3' UTR sequences and structures, conducting experimental analyses of RNA secondary structures using targeted and genome-wide DMS-MaPseq, evaluating transcript stability, performing bioinformatic analyses, correlating the acquired data with publicly available datasets, and ultimately experimentally validating the proposed model by engineering the 3' UTR of the FT gene.

Overall, the manuscript is profoundly intriguing and holds high scientific value. It appears to describe a phenomenon conserved among eukaryotic organisms. The authors reanalyzed publicly available data from rice and humans, arriving at conclusions similar to those drawn from Arabidopsis. Moreover, the presented results offer practical applications in designing specific 3' UTRs for generating transgenic plants with increased gene expression.

Nonetheless, certain aspects of the manuscript are unclear and require refinement prior to acceptance. Below, I have provided specific comments. It is important to reiterate that, on the whole, the manuscript holds substantial interest for me, and I believe it will captivate a broader audience within the Genome Biology journal.

The pri-miR159a in Arabidopsis seems to be properly transcribed and leads to the generation of miR159a (e.g. https://doi.org/10.3389/fpls.2022.905264). Thus, it is not clear to me why its transcription is blocked before the stem-loop structure, as in the PCHR2-LUC-pri-miR159a-3' UTR construct. What would cause the premature termination of the Pol? The authors should at least discuss that issue.

Could the selection of the CHR2 promoter and especially the 3'UTR somehow be related to the termination? Was there any specific purpose for using the CHR2 promoter and 3'UTR in constructs to drive LUC expression?

It is important to stress that DMS reactivity is related not only to the RNA's structure but also to the proteins binding to the RNA. Low DMS reactivity can suggest that the region is part of structured RNA or is bound by a protein. Thus, a properly designed experiment should also include in vitro folded RNA without proteins. I also miss the presentation of the raw DMS reactivities of the analyzed UTRs (using e.g. bar plots) in the supplementary data. Providing such plots for each biological replicate would make it easier to analyze the consistency of the obtained data, as the currently presented correlation plots (in 3D) are difficult to interpret. At this point, I strongly suggest avoiding 3D graphs (S2a and S4c) and replacing them with classical 2D plots for pair-wise comparison.

The authors looked for AU-rich, CCUC, and CUGC elements in high-Gini and low-Gini pools but were unable to detect any bias within these two categories. This is interesting and might suggest the presence of new motifs related to protein binding. Thus, I suggest using software like MEME to identify new DNA motifs that could be enriched in one of these two pools.

The authors should provide the results of 3'RACE for constructs presented in figure S3, as it is currently unclear if they modeled structures of the correct RNA fragments.

I would also consider changing the title of the manuscript, as DMS-MaPseq was not the only experiment to support the hypothesis.

The authors should also improve the readability of the figures by adding new information and more detailed descriptions in the captions. Here are some points I would like to highlight:

In figure 1a, where the schemes of constructs are presented, it is not clear what the colors represent, especially the red and gray lines. Initially, I thought that in the pri-miR159a-T1 construct, the red region was removed compared to pri-miR159a. However, this interpretation doesn't align with the presence of a transcription stop site in the red region, as shown in Figure S1. Therefore, it seems that the red color indicates the retained region in the construct, while the gray region is removed. This confusion persists when analyzing the schemes in figure S3, where the red color appears to indicate removed regions in the pri-miR159a-T2-1 and pri-miR159a-T2-2 constructs. Clear descriptions in the captions would resolve this ambiguity.

Lines 96-97: The sentence "To our big surprise, inclusion of pri-miR159a in the 3' UTR substantially increased, rather decreased, the expression of mRNA" is confusing due to the placement of "rather decreased." It can be rephrased as follows: "To our surprise, inclusion of pri-miR159a in the 3' UTR substantially increased mRNA expression, rather than decreasing it."

Line 123-124: It is stated that "inclusion of pri-miR159a in the 3' UTR substantially increased the accumulation of LUC mRNA and luminescence, by more than ten-fold." Based on the boxplots in figure 1b, the observed increase is estimated to be much lower than ten-fold. Evaluating from the medians, the LUC signal of 3'UTR is approximately 10,000, while the LUC signal in pri-miR159a-3' is 35,000, suggesting about a 3-fold increase.

Line 138 and Figure S1a: It is advisable to include quantification of the signal in different genotypes to verify that there is no significant difference in LUC signals.

Lines 178-180: Please include quantitative luminescence data for analyzed lines in Figure S1c. Adding a small panel showing the location of the ORFs within pri-miR159a would also be helpful.

Additionally, a graph with the construct and marked regions analyzed by ChIP-qPCR should be added to panel S1d. Currently, it is difficult to determine which regions were actually analyzed.

What was the folding energy of the artificially introduced stem-loop (PCHR2-LUC-stem-loop-3' UTR) compared to the self-folding pri-miR159a?

The quantitative data of LUC activity in a PCHR2-LUC-stem-loop-3' UTR construct should be presented.

Line 239: Replace "significant" with "significantly."

Line 401: "Addition" would be more appropriate than " Appendix".

Line 682: The provided link is not valid.

Figure S2c: Clarify how this correlation was calculated, i.e., whether DMS reactivities extracted from [25] were correlated with obtained reactivities.

Figure 3: Provide half-life values. Panel B is challenging to read. Dashed lines in the legend are difficult to distinguish from solid lines. Additionally, point sizes could be increased for better readability.

What was the read depth in DMS experiments for the analyzed regions?

A link or reference to the LightField software should be provided.

In Figure 1c, the loading control suggests unequal RNA loading on the gel.

Panels in figures are marked with small letters, while the legends use capital letters.

In Figure 2c, quantification of the luciferase signal should be presented alongside the pictures, or possibly in place of the pictures.

What was the number of human genes analyzed in figure 4f? The shapes of the red and blue lines suggest that a limited number of genes were analyzed, which could lessen the impact of the observation.

Line 147: Correct "Truncation1" to the appropriate term.

**Authors Response**

**Point-by-point responses to the reviewers’ comments:**

Reviewer reports:

Reviewer #1:

The manuscript elucidates an inverse correlation between the 3' UTR RNA secondary structure and transcript accumulation in Arabidopsis. The authors incidentally discovered that the pri-miR159a embedded in the 3' UTR of CHR2 can lead to the accumulation of the harbored transcript. They further showed that poorly structured 3' UTR enhanced mRNA stability, resulting in an increased accumulation of transcripts in vivo. Whereas the highly structured 3' UTR tend to associate lower expression levels and a shorter half-life compared to their counterparts with poorly structured 3' UTR. Additionally, the authors engineered 3' UTR with different structures into an inherent FT gene and manipulated the flowering time in Arabidopsis. This work demonstrated the regulation of 3' UTR structure on gene expression level and offers a new strategy to modify plant traits in agricultural production and mRNA stability by engineering the 3' UTRs RSS in biotechnology. Nevertheless, there are a few questions I think the author need to address to improve the manuscript.

1. Figure 1a. The author should use the same CCD camera to get the same background for PCHR2-LUC-pri-miR164a-3'UTR and its control as the others. All images should be taken with the same CCD camera and ensure the consistency in both the camera settings and the imaging conditions. Figure 2c has a similar issue.

**Response: We thank the reviewer for this criticism. We should have presented the figures 1a and 2c and their legends in a clearer way.**

**Briefly, there were two sets of experiments presented in the original Fig. 1a. One set experiment was performed much earlier via an older version of CCD camera, whereas the other was done later on via an upgraded CCD camera system. In each LUC assay, we always captured LUC images using the same CCD camera and with the same parameters. In addition, we compared the LUC intensity of different lines to their own control (CK, *P_CHR2_-LUC-3’ UTR*) to ensure consistency in each experiment.**

**For a clearer and more precise presentation of these data, we now re-organized the data by separating the images of CCD cameras into two different panels according to different experiments and different CCD cameras used in the revised version. In detail, we split the original Fig. 1a into new Fig. 1a and Fig. 1b. In the new Fig. 1a, LUC images of *P_CHR2_-LUC-3’ UTR*, *P_CHR2_-LUC-pri-miR159a-3’ UTR*, and *P_CHR2_-LUC-pri-miR164a-3’ UTR* were simultaneously captured using the same CCD camera and parameters. In the new Fig. 1b and subsequent experiments, the LUC images were grouped as they were captured with the new CCD camera. Furthermore, we added boxplots showing the quantification of the luciferase signal alongside the pictures. We also added one sentence in the figure legend “LUC pictures in (a) and (c) were taken under a CCD camera different from the one used in (b), with each experiment having its own CK (*P_CHR2_-LUC-3’ UTR*) lines.”.**

**Similarly, we separated the original Fig. 2c into new Fig. 2c and Fig. 2d in the revision for the precise presentation.**

**Fig. 1** Insertion of *MIR159a* in 3’ UTR enhances the expression of transgene. **a-b** LUC signals of reporter lines expressing different constructs. (Left part) schematic constructs. *P_CHR2_*, the native promoter of *CHR2* locus; *Nos*, nopaline synthase terminator. For different truncated segments of pri-miR159a, the red lines represented the retained regions of pri-miR159a, while the gray regions were removed in the constructs. (Middle part) Six-day-old T2 seedlings of 16 randomly selected independent lines were photographed under charge-coupled device (CCD) camera for LUC signals. The signal was displayed by LightField software. Exposure time for CCD camera was 30 S. (Right part in (**a**) and lower part in (**b**)) Quantification of luminescence results from different transgene lines. Each data point represented the mean of 10-12 plants from an individual lines. For most constructs,16 individual lines were utilized except for *P_CHR2_-LUC-stem-loop-3’ UTR* where only 9 lines were available. Statistical test was performed between different transgenic lines and *P_CHR2_-LUC-3’ UTR*. ns, no significance; **P* < 0.05; ***P* < 0.01; ****P* < 0.001; unpaired two-tailed Student’s *t*-test. **c** RNA blot analyses of randomly selected lines showed *LUC* transcript significantly accumulated in *P_CHR2_-LUC-pri-miR159a-3’ UTR*, but not *P_CHR2_-LUC-pri-miR164a-3’ UTR*, compared to *P_CHR2_-LUC-3’ UTR*. Ribosomal RNAs serve as control. The LUC signals of the sampled materials were shown in the bottom panels (Exposure time of 30 S under CCD camera). The relative signals of *LUC* blot were first normalized to that of *rRNAs*, and then to that of #1 of *P_CHR2_-LUC-3’ UTR* sample where the ratio was arbitrarily assigned a value of 1.0.

Be noted that LUC pictures in (**a**) and (**c**) were taken under a CCD camera different from the one used in (**b**), with each experiment having its own CK (*P_CHR2_-LUC-3’ UTR*) lines.

**Fig. 2c-d** RSS of the 3’ UTRs for different transgene transcripts. The DMS signal of A and C residues were color-coded and U/G bases were marked in gray. Quantification of luminescence results of the representative samples was shown in the right part. LUC pictures in (**c**) and (**d**) were taken under different CCD cameras, with each experiment having its own CK (*P_CHR2_-LUC-3’ UTR*) lines. Statistical test was performed between different transgenic lines and *P_CHR2_-LUC-3’ UTR*. ns, no significance; **P* < 0.05; ***P* < 0.01; ****P* < 0.001; unpaired two-tailed Student’s *t*-test.

2. Line285. How did the author demonstrate the success of the infiltration of IVT RNA? I did not find any evidence in the manuscript. The author used in vitro transcribed LUC fragment as a reference transcript to assess infiltration efficiency. However, they did not provide evidence of the expression of LUC protein. They should provide a figure of LUC signals photographed under CCD camera.

**Response: Thanks for this very insightful comment.**

**To demonstrate the success of the *IVTs* infiltration into seedlings, we re-did the experiments in a more quantitative way: Briefly, equal amounts of *in vitro* transcripts were either infiltrated (under vacuum conditions for 15 minutes) or left un-infiltrated (soaked for 15 minutes) in 10-day-old Col-0 seedlings. After treatment, plants were then washed three times in ddH_2_O before sampling to remove the potential remaining transcripts that might be stuck on the surface of the plants. Clearly, qRT-PCR assays showed that *in vitro* transcripts were negligibly detected in the un-infiltrated samples whereas significantly accumulated in the infiltrated samples. Conversely, the amount of *in vitro* transcripts leftover in the incubation solution was significantly higher in un-infiltrated samples compared to that of infiltrated samples (new Additional file 1: Fig. S3d). Thus, these results clearly validated the success of the *IVTs* infiltration and thereby strengthened the conclusion.**

**Of note, since our goal is to determine whether the 3’ UTR alone was enough to trigger structure-mediated RNA decay (SRD) [1], we performed *in vitro* transcription of the 3’ end regions of *P_CHR2_-LUC-3’ UTR* and *P_CHR2_-LUC-pri-miR159a-T1-3’ UTR*. These *IVTs* only contain 3’ end of LUC and its 3’ UTR regions, without 5’ UTR, start codon, and 5’ end of LUC regions). The *IVTs* did not cover the intact ORF. Consequently, we could not conduct western blot analysis of LUC proteins nor capture the LUC signals under CCD camera.**

**New Additional file 1: Fig. S3d** qRT-PCR assays validated the delivery efficiency of *in vitro* transcripts into plant tissues via vacuum infiltration. (Left panel) A significantly higher level of *in vitro* transcripts was detected inside the infiltrated samples vs the un-infiltrated samples. (Right panel) A significantly reduced amount of leftover *in vitro* transcripts was detected in the incubation solution and subsequent washing solution for the infiltrated samples vs the un-infiltrated ones. Equal amounts of *in vitro* transcripts were either infiltrated (under vacuum conditions for 15 minutes) or left un-infiltrated (soaked for 15 minutes) in 10-day-old Col-0 seedlings. After the treatment, plants were then washed three times in ddH_2_O before sampling. Relative *in vitro* transcripts amount in different treatments in Col-0 was normalized to that of *UBQ10*. *In vitro* transcripts leftover in the incubation solution, in 1^st^ washing ddH_2_O, and in 3^rd^ washing ddH_2_O were normalized to the amount in the initial incubation solution. The data were presented as means ± SE (n = 3) biologically independent replicates.

**More importantly, inspired by the insightful criticism by this reviewer, we tested our model directly using the stable transgenic lines. Briefly,** **we crossed *P_CHR2_-LUC-3’ UTR* and *P_CHR2_-LUC-pri-miR159a-T1-3’ UTR* lines with *sov* and *xrn4* mutants, respectively. The segregation in F2 population allowed us to concurrently obtain the stable lines of *P_CHR2_-LUC-3’ UTR* and *P_CHR2_-LUC-pri-miR159a-T1-3’ UTR* in the mutants (*sov* and *xrn4*) and Col-0 backgrounds. Using these stable homozygote lines, we did a new round of RNA decay assay. Again, we observed that the significant difference of *LUC* decay rates of *P_CHR2_-LUC-3’ UTR* and *P_CHR2_-LUC-pri-miR159a-T1-3’ UTR* in Col-0 background was abolished in both *sov* and *xrn4* mutants, reminiscent of the results from semi *in vitro* decay assays. These new results further boosted our model that transcripts with highly structured 3’ UTRs are degraded faster than the ones with poorly structured 3’ UTRs, and the decay is through both SOV and XRN4 pathways. In the revised version, we added the new decay data in stable mutants (*sov* and *xrn4*) in new Fig. 3c (lines 316-327).**

**New Fig. 3c** qRT-PCR showed that the decay of transcripts with highly structured 3’ UTRs is through SOV and XRN4. 10-day-old seedlings were treated with 50 μM Act D for indicated times before sampling. The relative expression of *LUC* was normalized to that of *18S rRNA*.

3. Figure 3. How to explain the accumulation of the transcripts of pri-miR159a-T1-3'UTR after 1h infiltration in 3c. As the RNA is transcribed in vitro, its abundance cannot increase after infiltration. In addition, in the right panel of 3a, the error bar of pri-miR159a-T1-3'UTR at 0 h is too high.

**Response: We thank the reviewer for this comment.**

**For accumulation of the *in vitro* transcripts of *pri-miR159a-T1-3’ UTR* after 1h infiltration, firstly, we interpreted it as the variation of biological replicates. In fact, similar variations have been often observed in *in vivo* conditions. For instance, the transcript levels remained relatively higher even after 100 min of transcription inhibitor treatment [2, 3]. In our hands, we also performed *t*-test and found no significant differences in transcript levels among 0, 0.5 h, and 1 h treatments. The results suggested that the *in vitro* transcripts of *pri-miR159a-T1-3' UTR* exhibited significant stability, showing minimal degradation even after 1 hour of infiltration. Our new *in vivo* decay data in exoribonuclease mutants (*sov* and *xrn4*) vs Col-0 further substantiated and reinforced this conclusion (New Fig. 3c) in lines 316-327 in the revision.**

**In addition, in the right panel of 3a, the elevated error bar for pri-miR159a-T1-3’ UTR at 0 h was also considered as the variation of biological replicates.**

4. L329-L344. It is hard to understand the higher expression of genes with poorly structured 3' UTR are involved in stress-related pathways. Since the gene expression data is obtained from Col-0 plants, other than stressed plants, the genes involved in stress-related pathway should not be induced and therefore should not display a high expression level.

**Response: We thank the reviewer for this comment. We carefully examined the expression levels of stress-related genes in this study via published RNA-seq data [4, 5]. Our analysis showed that the genes involved in “response to light intensity” and “response to salt stress” exhibit rather high expression levels (most of them are more than 1 RPKM) in Col-0. Almost all these genes do not significantly respond to light or salt treatment. This result suggests that these stress-related genes are already maintained in high expressed levels, instead of being induced under stress. Please refer to the figures below for more details.**

5. Figure 3b. It is hard to distinguish the label of three groups lines, which is not consistent with the line chart, especially for the Col-0 (solid line) and sov (half dashed line).

**Response: Thanks for the comment. We revised Fig. 3b for better readability.**

**Fig. 3b** qRT-PCR showed the relative mRNA abundance of *in vitro* transcribed 3’ end region of *LUC* transcripts of *P_CHR2_-LUC-3’ UTR* (*CK-3’ UTR*) and *P_CHR2_-LUC-pri-miR159a-3’ UTR* (*pri-miR159a-T1-3’ UTR*) delivered into Col-0, *sov*, and *xrn4*. The same level of *in vitro* transcripts was infiltrated into 10-day-old Col-0, *xrn4*, and *sov* seedlings, respectively. Infiltrated plants were collected at indicated time points for qRT-PCR. A different *in vitro* transcribed segment of *LUC* transcript was co-infiltrated as a reference for normalization.

6. In Method section, almost all the plasmids contained a "FM" element which is not appear in the main text. What did it indicate?

**Response: Thanks for the comment. “FM” indicates ‘Flag-4Myc’. The plasmids contain the FM elements. We corrected this typo in the revision (line 473).**

Reviewer #2: Summary:

The study investigates the impact of RNA secondary structure (RSS) within 3' untranslated regions (3' UTRs) on mRNA stability and gene expression in Arabidopsis. It reveals that highly structured 3' UTRs lead to mRNA destabilization, while poorly structured 3' UTRs enhance mRNA accumulation. The research also indicates that this pattern extends from plants (Arabidopsis and rice) to mammals (human). The study suggests that manipulating 3' UTR RSS could be a potential strategy to modify gene expression and plant traits, offering implications for both agricultural production and biotechnology.

Overall Evaluation:

The presented study challenges the conventional understanding of the roles of RNA secondary structure (RSS) within 3' UTRs and offers insights into their influence on mRNA stability and gene expression. However, several aspects of the study should be further addressed before considering it for publication.

Detailed Comments:

1. Genomic Analysis and Confounding Factors:

The genomic analysis (Figures 4 and 5) constitutes only a smaller proportion of this study. The genomic analysis lacks consideration of potential confounding factors, which could impact the reported correlations. Controlling for such factors would strengthen the study's conclusions and improve the validity of the presented associations. Transcript properties are often associated with each other among genes to some degree, but many disappear when confounding factors are controlled for.

**Response: Thanks so much for sharing this great idea.**

**In the original manuscript, we examined various confounding factors, including 3’ UTR length, RG4 structure, AU-rich element, CCUC motif, and CUGC motif. These factors were determined not to be implicated in the correlation between 3’ UTR RSS and gene expression (previous Additional file 1: Fig. S4h, i were re-organized to the new Additional file 1: Fig. S5f, g).**

**Following this reviewer’s advice, we further expanded our investigation to include GC content, poly(A) tail length, and miRNA targeting in the revised version. Notably, 3’ UTR transcripts with high-Gini were observed to possess lower GC content and longer poly(A) tail length compared to 3' UTR transcripts with low-Gini. This aligns with existing conclusions that transcripts with lower GC content and longer poly(A) tail length tend to be less stable [6, 7]. Subsequently, we also re-analyzed this correlation after controlling for GC content and poly(A) tail length. Interestingly, we found that when GC content (high-GC transcripts) or poly(A) tail length (both for long poly(A) and short poly(A) transcripts) was maintained at a similar level, low-Gini genes still exhibited higher expression levels (new Additional file 1: Fig. S5d, e). Regarding miRNA targeting, we did not detect a significant difference in the distribution of targeting sites between high-Gini and low-Gini 3’ UTRs (new Additional file 1: Fig. S5h). This makes sense that 3’ UTRs of transcripts are not the hotspots targeted by plant miRNAs as otherwise observed in metazoan miRNAs [8]. In summary, the identified negative correlation between 3’ UTRs RSS and transcript expression levels is not confounded by any of the tested factors such as AU-rich element, CCUC motif, CUGC motif, GC content, poly(A) tail length, 3’ UTR length, RG4, or miRNA targeting (lines 381-421).**

**New Additional file 1: S5d** (Left panel) Comparison of 3’ UTR GC content (%) between high-Gini vs low-Gini genes. (Middle panel) Genes were categorized into high and low GC content, with half of the genes having GC content ≥ 32.55% and half having < 32.55%. Boxplots were used to compare the difference of GC content between high-Gini genes and low-Gini genes. (Right panel) Comparison of gene expression level (RPKM) between high-Gini and low-Gini genes in two different GC content categories. *P* values by Wilcoxon test. **e** (Left panel) Comparison of poly(A) tail length between high-Gini vs low-Gini genes. (Middle panel) Genes were classified into long and short poly(A) tail length, with half of the genes having poly(A) tail length ≥ 80 and half having < 80 nt. Boxplots were used to compare the difference in poly(A) tail lengths between high-Gini genes and low-Gini genes. (Right panel) Comparison of gene expression level (RPKM) between high-Gini and low-Gini genes in two different poly(A) tail length categories. *P* values by Wilcoxon test. Poly(A) tail length data was acquired from previous publication [6]. **f** 3’ UTR length between high-Gini vs low-Gini genes. *P* values by Wilcoxon test.

2. Data Presentation and Interpretation:

The classification of genes into categories in Figure 4c, e, f (and Figure 5a, b) appears arbitrary, making it difficult to evaluate the proportion of variation in mRNA levels that can be attributed to mRNA secondary structure. Encouraging the inclusion of scatter plots and correlation analysis would enhance the clarity and robustness of the presented data. In figure 5a, it is also not clear why ks test was used only for part of data.

**Response: We thank the reviewer for this excellent idea. We have included the scatter plots in the revised version in the new Additional file 1: Fig. S5a-c, S6b-d.**

**The scatter plots between 3’ UTRs RSS (Gini index values) and transcripts expression levels (RPKM) show a negative correlation in *Arabidopsis*, rice, and human (new Additional file 1: Fig. S5a-c) (lines 362-364 and lines 375-377).**

**The negative correlations between 3’ UTRs RSS (average Gini) and RNA stability (RNA half-life) are also significant in both RNA-decay datasets [2, 3]. Moreover, we found that the transcripts with 3’ UTR Gini index exceeding 0.4 exhibit a more significantly negative correlation, which further demonstrated that once RSS of transcripts exceeds a high threshold (like Gini index ≥ 0.4), the mRNA tends to be unstable (new Additional file 1: Fig. S6b-c) (lines 437-440).**

**A positive correlation exists between 3’ UTRs RSS (average Gini) and RNA half-lives fold change (*sov*/WT). This intriguing result means that SOV may preferentially bind to transcripts with highly structured 3' UTRs *in vivo*. Essentially, this finding suggests that SOV is implicated in the decay of the more structured transcripts (new Additional file 1: Fig. S6d) (line 448).**

**New Additional file 1: Fig. S5a-c** Scatter plots of correlation analysis between 3’ UTR RSS (average Gini) and RNA expression level (log_10_(RPKM)) in *Arabidopsis* (**a**), rice (**b**), and human (**c**). Be noted that RSS of 3’ UTRs was decoded through a TGIRT-mutational profiling method in *Arabidopsis* and human but through a regular reverse transcription-stop method in rice. *R*, Pearson’s correlation coefficient. *P* was calculated by Pearson’s correlation analysis.

**New Additional file 1: S6** RSS of 3’ UTRs is inversely correlated with transcripts half-life. **a** Comparison of Gini indexes between short and long half-life genes in *Arabidopsis* (RNA decay data, GSE136713). *P* value by Kolmogorov-Smirnov test. **b-c** Scatter plots of correlation analysis between 3’ UTRs RSS (average Gini) and RNA half-life (log_10_(half-life min)) of published data GSE86361 (**d**) and GSE136713 (**e**). *R*, Pearson’s correlation coefficient. *P* was calculated by Pearson’s correlation analysis. **d** Scatter plot of correlation analysis between 3’ UTRs RSS (average Gini) and RNA half-lives fold change (*sov*/WT) in *Arabidopsis* (RNA decay data, GSE86361). *R*, Pearson’s correlation coefficient. *P* was calculated by Pearson’s correlation analysis. **e** Motifs enriched by MEME for high-Gini and low-Gini 3’ UTRs.

**In Fig. 5a, we performed ks test both for all high-Gini and low-Gini transcripts (*P* = 0.045) and transcripts with Gini ≥ 0.4 (*P* = 0.028). We labeled the *P* value in Fig. 5a left part in revised Figures.**

3. Presentation Structure and Focus:

The study starts with an unexpected observation regarding pri-miR159a, which may confuse readers about the study's broader implications and its relevance to RNA secondary structure (RSS) within 3' UTRs in other sequence contexts. Providing a more coherent context and clearly establishing the relevance of the observed phenomenon would improve the study's overall structure.

**Response: We thank the reviewer for this comment.**

**We have now provided a more detailed context to make it more readable and coherent. In the introduction (line 94) and results parts (lines 115-116), we explained and described that pri-miRNAs are featured with a hairpin folded structure to emphasize the relevance to RSS within 3’ UTRs.**

4. Suggested Additional Analysis:

Related to the previous comment, considering the controversies in the field, the incorporation of Massively Parallel Reporter Assays could provide a robust validation of the findings and help resolve uncertainties related to the role of RNA secondary structure (RSS) in mRNA stability and gene expression.

**Response: We thank the reviewer for this insightful idea.**

**Massively Parallel Reporter Assays (MPRAs) offer a high-throughput approach to assess the functional impact of genetic elements on gene expression by simultaneously analyzing thousands to millions of DNA sequences in a single experiment. Incorporating MPRAs would enhance the reliability and comprehensiveness of experimental data, providing valuable insights into the intricate relationship between RSS and gene regulation. This idea holds promise for future research on uncertainties regarding the role of RSS in mRNA stability and gene expression, but clearly is beyond the scope of this study.**

5. 3'-UTR Terminology:

There is a misunderstanding in the terminology used for 3' UTRs throughout the manuscript. The study refers to the "end" of 3' UTRs as the point of transcription termination, whereas it should be clarified that it is the site of polyadenylation.

**Response: We thank the reviewer for this comment. We corrected the terminology in the revision (lines 36, 99, 162, 205-215, 278, 485, 576, and 690-691).**

In conclusion, the study presents intriguing insights into the relationship between RNA secondary structure (RSS) within 3' UTRs and mRNA stability across various organisms. However, addressing the outlined concerns and refining the presentation will be crucial in enhancing the validity and impact of the findings.

Reviewer #3: The manuscript titled "DMS-MaPseq Implicates Structured 3' UTRs in mRNA Destabilization in Plants," authored by Zhang and colleagues, reveals that nuclear transcripts with weak secondary structures in their 3' UTRs tend to exhibit greater stability compared to those with highly structured 3' UTRs, thereby promoting gene expression.

Zhang et al. present compelling experiments, including generating transgenic plants with modified 3' UTR sequences and structures, conducting experimental analyses of RNA secondary structures using targeted and genome-wide DMS-MaPseq, evaluating transcript stability, performing bioinformatic analyses, correlating the acquired data with publicly available datasets, and ultimately experimentally validating the proposed model by engineering the 3' UTR of the FT gene.

Overall, the manuscript is profoundly intriguing and holds high scientific value. It appears to describe a phenomenon conserved among eukaryotic organisms. The authors reanalyzed publicly available data from rice and humans, arriving at conclusions similar to those drawn from Arabidopsis. Moreover, the presented results offer practical applications in designing specific 3' UTRs for generating transgenic plants with increased gene expression.

Nonetheless, certain aspects of the manuscript are unclear and require refinement prior to acceptance. Below, I have provided specific comments. It is important to reiterate that, on the whole, the manuscript holds substantial interest for me, and I believe it will captivate a broader audience within the Genome Biology journal.

The pri-miR159a in Arabidopsis seems to be properly transcribed and leads to the generation of miR159a (e.g. https://doi.org/10.3389/fpls.2022.905264). Thus, it is not clear to me why its transcription is blocked before the stem-loop structure, as in the PCHR2-LUC-pri-miR159a-3' UTR construct. What would cause the premature termination of the Pol? The authors should at least discuss that issue.

Could the selection of the CHR2 promoter and especially the 3'UTR somehow be related to the termination?

**Response: We thank the reviewer for this comment. We discussed the potential mechanisms in lines 516-523 in the revised version.**

**In the article (e.g.** [**https://doi.org/10.3389/fpls.2022.905264**](https://doi.org/10.3389/fpls.2022.905264)**), pri-miR159a could be properly transcribed as a gene with its native promoter. However, in our cases, we introduced pri-miR159a in the 3’ UTR region. Our 3’ RACE experiment and 3’ end target-specific DMS-MaPseq identified the polyadenylation sites of the transcripts, not the transcription termination sites. In *P_CHR2_-LUC-pri-miR159a-3’ UTR* transgenic line, its polyadenylation (poly(A) site) is at the 5’ end segment of pri-miR159a, rather than extending through the hairpin structure region to reach the very 3’ end of their 3’ UTRs. The early polyadenylation might be a consequence of the poly(A) site determination.**

**Polyadenylation site determination is a complex process involving the intricate interplay between *cis*-elements within pre-mRNA and the cleavage and polyadenylation molecular complex (CPMC). In mammals, the pivotal role of the hexamer A(A/U)UAAA as a polyadenylation signal (PAS) in influencing the exact location of the polyadenylation site is well-documented. In plants, a tripartite model for polyadenylation signal is proposed, comprising the far upstream element (FUE), the near upstream element (NUE), and the cleavage element (CE). FUE (6-18 nt) is composed of a region rich in U>A>G, NUE (6-10 nt) is A-rich, and CE is rich in U>A>C. Unlike mammals, mutations in *cis*-elements minimally affect polyadenylation efficiency; some may even enhance functionality of *cis*-elements, indicating lower conservation of PAS *cis*-elements in plants [9]. In our study, introducing sequences into *CHR2* 3’ UTR yielded transcripts with identical 5’ UTR and CDS but distinct 3’ UTR structures may harbor different polyadenylation signals. Despite precautions against A(A/U)UAAA inclusion, inadvertent introduction of unexpected *cis*-elements into 3’ UTR regions, leading to premature polyadenylation, is possible.**

**Beyond the inadvertent inclusion of *cis*-elements in the 3’ UTR, DNA sequences and structure may also influence the choice of the poly(A) site. It is well-established that certain DNA sequences and structures, as well as RNA structure, could induce Pol II pausing, resulting in transcription termination and contributing to the usage of poly(A) site [10]. Thus, in our study, early polyadenylation may also be attributed to unexpected and intricate DNA or RNA structures arising from the introduction of additional sequences into the 3’ UTR region.**

**One literature indicates a global influence of transcription initiation sites (TSS) on the choice of 3’ end sites [11]. Specifically, certain promoters characterized by deficient H2A.Z and abundant H3K18Ac can be recognized by CBP molecules. These CBP molecules also interact with specific poly(A) sites, facilitating the linkage between the 5’ TSS and 3’ end processing. It is noteworthy that this promoter feature is typically observed in genes exhibiting endogenous alternative TSS usage. Therefore, it remains a possibility that the artificial construct with *CHR2* promoter could exert similar effects on poly(A) site determination for *LUC* transcripts.**

Was there any specific purpose for using the CHR2 promoter and 3'UTR in constructs to drive LUC expression?

**Response: Thanks for the comment. Initially, we wanted to study the function of Microprocessor, so we introduced the LUC reporter in the context of *CHR2* gene [12, 13] (lines 120-125).**

It is important to stress that DMS reactivity is related not only to the RNA's structure but also to the proteins binding to the RNA. Low DMS reactivity can suggest that the region is part of structured RNA or is bound by a protein. Thus, a properly designed experiment should also include in vitro folded RNA without proteins.

**Response: Thanks for this reviewer’s insightful suggestion.**

**Per the advice, we have now performed *in vitro* 3’end target-specific DMS-MaPseq for the *in vitro* folded RNA. The 3’ UTRs of high *LUC* expression lines (*P_CHR2_-LUC-pri-miR159a-3’ UTR* and *P_CHR2_-LUC-pri-miR159a-T1-3’ UTR*) still showed less-structured compared with the low *LUC* expression lines (*P_CHR2_-LUC-3’ UTR* and *P_CHR2_-LUC-pri-miR164a-3’ UTR*), which is consistent with the *in vivo* 3’end target-specific DMS-MaPseq results. We added the results in new Fig. 2f during this revision (lines 254-264). We conclude that the higher Gini indexes observed in the 3’ UTRs of *LUC* transcripts in the low expression lines *in vivo* are due to their intrinsic RSS, rather than the hindrance of DMS modification by protein binding. This result is also in line with other reports that the association of the proteins might only have negligible effects on DMS reactivity [14].**

**New Fig. 2f** Gini index of *in vitro* DMS reactivities of the 3’ UTRs from different transgene lines. *P* values by Wilcoxon test.

I also miss the presentation of the raw DMS reactivities of the analyzed UTRs (using e.g. bar plots) in the supplementary data. Providing such plots for each biological replicate would make it easier to analyze the consistency of the obtained data, as the currently presented correlation plots (in 3D) are difficult to interpret. At this point, I strongly suggest avoiding 3D graphs (S2a and S4c) and replacing them with classical 2D plots for pair-wise comparison.

**Response: Thanks for the comment. We also analyzed the biological replicates’ correlation with classical 2D plots as follows. The 2D plot might simplify data visualization, while we recognize that a 3D plot could potentially offer a more detailed and nuanced view of the relationships among three biological replicates in a rather compact manner. Moreover, the 3D plots provide us with additional insights and perspectives compared to traditional 2D plots. Therefore, we have opted to continue using 3D plots in our revision.**

The authors looked for AU-rich, CCUC, and CUGC elements in high-Gini and low-Gini pools but were unable to detect any bias within these two categories. This is interesting and might suggest the presence of new motifs related to protein binding. Thus, I suggest using software like MEME to identify new DNA motifs that could be enriched in one of these two pools.

**Response: We thank the reviewer for this good idea. We used MEME to uncover potential new DNA motifs. U-rich and A-rich motifs were identified in high-Gini 3’ UTRs, and CU-rich and U-rich motifs were observed in low-Gini 3’ UTRs. The presence of U-rich motifs in both high-Gini and low-Gini categories could be a common characteristic of 3’ UTR regions. Simultaneously, A-rich motifs in high-Gini and CU-rich motifs in low-Gini categories could be associated with distinct protein binding patterns. Whether these motifs are *bona fide* elements and/or related to gene expression awaits future clarification. The data was presented in the new Additional file 1: Fig. S6e (lines 512-515).**

**New Additional file 1: Fig. S6e** Motifs enriched by MEME for high-Gini and low-Gini 3’ UTRs.

The authors should provide the results of 3'RACE for constructs presented in figure S3, as it is currently unclear if they modeled structures of the correct RNA fragments.

**Response: Thanks for the comment. In the revision, we added the 3’ RACE results for each construct presented in new Additional file 1: Fig. S3a (lines 277-279).**

**New Additional file 1: Fig. S3a** 3’ RACE experiment identified poly(A) sites of *LUC* transcripts from different transgenic lines. The poly(A) sites of *P_CHR2_-LUC-pri-miR159a-T2-3’ UTR*, *P_CHR2_-LUC-stem-loop-3’ UTR*, and *P_CHR2_-LUC-pri-miR159a-T2-2-3’ UTR* were sequenced by Sanger sequencing, and *P_CHR2_-LUC-pri-miR159a-T2-1-3’ UTR* was identified from high throughput sequencing.

I would also consider changing the title of the manuscript, as DMS-MaPseq was not the only experiment to support the hypothesis.

**Response: Thanks for the comment.**

**We changed the title to “Structured 3’ UTRs destabilize mRNAs in plants” (line 1).**

The authors should also improve the readability of the figures by adding new information and more detailed descriptions in the captions. Here are some points I would like to highlight:

In figure 1a, where the schemes of constructs are presented, it is not clear what the colors represent, especially the red and gray lines. Initially, I thought that in the pri-miR159a-T1 construct, the red region was removed compared to pri-miR159a. However, this interpretation doesn't align with the presence of a transcription stop site in the red region, as shown in Figure S1. Therefore, it seems that the red color indicates the retained region in the construct, while the gray region is removed. This confusion persists when analyzing the schemes in figure S3, where the red color appears to indicate removed regions in the pri-miR159a-T2-1 and pri-miR159a-T2-2 constructs. Clear descriptions in the captions would resolve this ambiguity.

**Response: We thank the reviewer for the comment. We should have explained our figures clearly. We revised the description and added the sentence in the figure legends in Fig. 1b and Additional file 1: Fig. S3b in the revision “For different truncated segments of pri-miR159a, the red lines represented the retained regions of pri-miR159a, while the gray regions were removed in the constructs.”.**

Lines 96-97: The sentence "To our big surprise, inclusion of pri-miR159a in the 3' UTR substantially increased, rather decreased, the expression of mRNA" is confusing due to the placement of "rather decreased." It can be rephrased as follows: "To our surprise, inclusion of pri-miR159a in the 3' UTR substantially increased mRNA expression, rather than decreasing it."

**Response: Thanks for the comment. We revised the description (lines 97-98).**

Line 123-124: It is stated that "inclusion of pri-miR159a in the 3' UTR substantially increased the accumulation of LUC mRNA and luminescence, by more than ten-fold." Based on the boxplots in figure 1b, the observed increase is estimated to be much lower than ten-fold. Evaluating from the medians, the LUC signal of 3'UTR is approximately 10,000, while the LUC signal in pri-miR159a-3' is 35,000, suggesting about a 3-fold increase.

**Response: We thank the reviewer for the comment. The 10-fold increase initially referred to the signal change in RNA blot. It seems like that the signal increase did not match well from RNA blot and CCD image. But we corrected our description in the revision in lines 126-128 as follows “We found that the inclusion of pri-miR159a in the 3’ UTR substantially increased the accumulation of *LUC* mRNA and luminescence, by more than 10- and three-fold compared to the control construct, respectively, in which pri-miR159a was deleted (CK, *P_CHR2_-LUC-3’ UTR*).”.**

Line 138 and Figure S1a: It is advisable to include quantification of the signal in different genotypes to verify that there is no significant difference in LUC signals.

**Response: Thanks for the comment. We added quantification results of the LUC signal in different genotypes in Additional file 1: Fig. S1a and there was no significant difference in LUC signals in different genotypes.**

**Additional file 1: Fig. S1a** LUC signals of T2 three-week-old transgenic plants (left panel). Quantification of luminescence results from different transgene lines (right panel). *dcl1-9* and *se-3* homozygotes (-/-) displayed small statues and were circled in red. Scale bar, 0.5 cm. Statistical test was performed among different transgenic lines. ns, no significance; unpaired two-tailed Student’s *t*-test.

Lines 178-180: Please include quantitative luminescence data for analyzed lines in Figure S1c. Adding a small panel showing the location of the ORFs within pri-miR159a would also be helpful.

**Response: Thanks for the comment. We added a graph showing the location of the ORFs within pri-miR159a and the quantification results of the LUC signal in Additional file 1: Fig. S1c.**

**Additional file 1: Fig. S1c** Double missense mutations of start codons for two hypothetic miPEPs in pri-miR159a-T1 did not alter the patterns of luminescence compared with *P_CHR2_-LUC-pri-miR159a-T1-3’ UTR*. The locations of ORFs predicted to decode two miPEPs were shown in schematics (upper panel). Quantification of luminescence results from different transgene lines (lower panel). Exposure time of LUC signals from CCD camera was 20 S. DM, double mutations. Statistical test was performed between different transgenic lines and *P_CHR2_-LUC-3’ UTR*. ns, no significance; **P* < 0.05; ***P* < 0.01; ****P* < 0.001; unpaired two-tailed Student’s *t*-test.

Additionally, a graph with the construct and marked regions analyzed by ChIP-qPCR should be added to panel S1d. Currently, it is difficult to determine which regions were actually analyzed.

**Response: Thanks for the comment. We added a graph with the constructs and marked regions analyzed by ChIP-qPCR in Additional file 1: Fig. S1d.**

**Additional file 1: Fig. S1d** ChIP–qPCR assay did not reveal significant change of RNA polymerase II occupancy in *CHR2* promoter (*P_CHR2_*) and *LUC* locus in different transgenic lines (*P_CHR2_-LUC-3’ UTR*, *P_CHR2_-LUC-pri-miR159a-3’ UTR*, and *P_CHR2_-LUC-pri-miR159a-T1-3’ UTR*). Regions selected for ChIP-qPCR analysis were marked in the graphs (left panel). ChIP signal was first normalized to input, and then to that of *UBQ10*. *UBQ10* serves as a positive control. ChIP without the antibody (no AB) served as negative controls. The data were presented as means ± SE (n = 3) biologically independent replicates.

What was the folding energy of the artificially introduced stem-loop (PCHR2-LUC-stem-loop-3' UTR) compared to the self-folding pri-miR159a?

**Response: The predicted folding energy of the artificial stem-loop is -20.1, whereas the self-folding pri-miR159a is -75.5. However, for *P_CHR2_-LUC-pri-miR159a-3' UTR*, its poly(A) site precedes the self-folding region, and its 3’ UTR folding energy is -35.6. For *P_CHR2_-LUC-stem-loop-3' UTR*, its poly(A) site is located in the 3’ UTR region, and its 3’ UTR folding energy is -72.7. Consequently, based on their folding energy, the transcript of *P_CHR2_-LUC-pri-miR159a-3' UTR* is less structured, aligning with our conclusion.**

The quantitative data of LUC activity in a PCHR2-LUC-stem-loop-3' UTR construct should be presented.

**Response: Thanks for the comment. The quantitative data of LUC activity in a *P_CHR2_-LUC-stem-loop-3’ UTR* construct was presented using a boxplot in the lower part of Fig. 1b.**

Line 239: Replace "significant" with "significantly."

**Response: Corrected (line 246).**

Line 401: "Addition" would be more appropriate than " Appendix".

**Response: Changed (line 466).**

Line 682: The provided link is not valid.

**Response: The link should work now (line 777).**

Figure S2c: Clarify how this correlation was calculated, i.e., whether DMS reactivities extracted from [25] were correlated with obtained reactivities.

**Response:** **We thank the reviewer for this comment. In 3’ end target-specific DMS-MaPseq and DIM-2P-seq, the secondary structures of nucleotides 121-186 of *CAB1* (At1g29930) mRNA were determined using DMS reactivities. DMS reactivities from gel-based RNA structure probing method were extracted [15]. Pearson correlation coefficient (PCC) of DMS reactivities was calculated between our study and the published paper [15]. Our *in vivo* DMS reactivities exhibited strong agreement with previously published data (PCC = 0.626 in 3’ end target-specific DMS-MaPseq in Additional file 1: Fig. S2c and PCC = 0.688 in DIM-2P-seq in Additional file 1: Fig. S4e).**

**We added the method in the figure legend in Additional file 1: Fig. S2c and Additional file 1: Fig. S4e in our revised version.**

Figure 3: Provide half-life values. Panel B is challenging to read. Dashed lines in the legend are difficult to distinguish from solid lines. Additionally, point sizes could be increased for better readability.

**Response: We thank the reviewer for this comment. In the revised version, we provided the half-life values of *LUC* and *F-Box* transcripts in Fig. 3a. We also re-drew Fig. 3b for better readability as follows.**

**Fig. 3a** qRT-PCR assays show the relative expression of *LUC* (left) and *F-box* (right) in different transgenic samples (*P_CHR2_-LUC-3’ UTR*, *P_CHR2_-LUC-pri-miR159a-3’ UTR*, and *P_CHR2_-LUC-pri-miR159a-T1-3’ UTR*) collected at indicated times after the treatment with 50 μM Act D. Half-life *t*_1/2_ is shown. *LUC_CK_* *t*_1/2_ < *LUC_159a/159a-T1_* *t*_1/2_. *F-box_CK_* *t*_1/2_ ≅ *F-box_159a/159a-T1_* *t*_1/2_. RNA was extracted from 10-day-old seedlings of each line. The relative expression of tested genes was normalized to that of *18S rRNA*. Data are shown as means ± SE from three independent biological replicates. ns, no significance; **P* < 0.05; ***P* < 0.01; unpaired two-tailed Student’s *t*-test. **b** qRT-PCR showed the relative mRNA abundance of *in vitro* transcribed 3’ end region of *LUC* transcripts of *P_CHR2_-LUC-3’ UTR* (*CK-3’ UTR*) and *P_CHR2_-LUC-pri-miR159a-3’ UTR* (*pri-miR159a-T1-3’ UTR*) delivered into Col-0, *sov*, and *xrn4*. The same level of *in vitro* transcript was infiltrated into 10-day-old Col-0, *xrn4*, and *sov* seedlings, respectively. Infiltrated plants were collected at indicated time points for qRT-PCR. A different *in vitro* transcribed segment of *LUC* transcript was co-infiltrated as a reference for normalization.

What was the read depth in DMS experiments for the analyzed regions?

**Response: Thanks for the comments. Based on the conclusion from Zubradt *et al.* [16], an average mismatch coverage depth of 20X was set as the threshold for the analyzed regions (lines 791 and 826-827).**

A link or reference to the LightField software should be provided.

**Response: Thanks for the comments. LightField software version and link are provided in the methods in the revised version in lines 656-657.**

**LightField Software Version: 6.4.1.1709.**

**link: https://www.princetoninstruments.com/products/software-family/lightfield.**

In Figure 1c, the loading control suggests unequal RNA loading on the gel.

**Response: Thanks for the comments. Because our initial CCD screening suggested a significantly lower level of LUC signal in the control line, we then purposely increased the loading amount of total RNA of *P_CHR2_-LUC-3’ UTR*. Such a design would enable us to detect the LUC signal from the plants expressing different constructs in the same blot.**

Panels in figures are marked with small letters, while the legends use capital letters.

**Response:** **Changed.**

In Figure 2c, quantification of the luciferase signal should be presented alongside the pictures, or possibly in place of the pictures.

**Response:** **Thanks very much for this comment. We have reorganized the data, separating distinct lines captured by different CCD cameras into two small panels. Additionally, we have added boxplots showing the quantification of the luciferase signal alongside the pictures.**

**Also, see the response to the question #1 of Reviewer #1.”.**

**Fig. 2c-d** RSS of the 3’ UTRs for different transgene transcripts. The DMS signal of A and C residues were color-coded and U/G bases were marked in gray. Quantification of luminescence results of the representative samples was shown in the right part. LUC pictures in (**c**) and (**d**) were taken under different CCD cameras, with each experiment having its own CK (*P_CHR2_-LUC-3’ UTR*) lines. Statistical test was performed between different transgenic lines and *P_CHR2_-LUC-3’ UTR*. ns, no significance; **P* < 0.05; ***P* < 0.01; ****P* < 0.001; unpaired two-tailed Student’s *t*-test.

What was the number of human genes analyzed in figure 4f? The shapes of the red and blue lines suggest that a limited number of genes were analyzed, which could lessen the impact of the observation.

**Response: Thanks very much for this comment. Upon revisiting the data, we noted that the original manuscript only incorporated data from one biological replicate. Subsequently, after merging two biological replicates data, we identified 129 low-Gini genes and 129 high-Gini genes. We have now redrawn and replaced the previous Fig. 4f. The updated figure displays smoother shapes for the red and blue lines, making the conclusion more robust and valid.**

**Fig. 4f** Comparison of gene expression level (RPKM) between high-Gini and low-Gini genes for human. *P* value < 2.2e^-16^ by Wilcoxon test.

Line 147: Correct "Truncation1" to the appropriate term.

**Response: Thanks for the comment. The description of “pri-miR159a Truncation1 line” is too general. We revised it to “the 5’ segment of pri-miR159a (1-274 nt) (159a-T1, *P_CHR2_-LUC-pri-miR159a-T1-3’ UTR*)” in lines 150-151 during the revision.**

1.	Fischer JW, Busa VF, Shao Y, Leung AKL. Structure-Mediated RNA Decay by UPF1 and G3BP1. Mol Cell. 2020,78:70-84.e76.

2.	Chantarachot T, Sorenson RS, Hummel M, Ke H, Kettenburg AT, Chen D, Aiyetiwa K, Dehesh K, Eulgem T, Sieburth LE, et al. DHH1/DDX6-like RNA helicases maintain ephemeral half-lives of stress-response mRNAs. Nat Plants. 2020,6:675-685.

3.	Sorenson RS, Deshotel MJ, Johnson K, Adler FR, Sieburth LE. Arabidopsis mRNA decay landscape arises from specialized RNA decay substrates, decapping-mediated feedback, and redundancy. Proc Natl Acad Sci U S A. 2018,115:E1485-E1494.

4.	Anderson SJ, Kramer MC, Gosai SJ, Yu X, Vandivier LE, Nelson ADL, Anderson ZD, Beilstein MA, Fray RG, Lyons E, et al. N(6)-Methyladenosine Inhibits Local Ribonucleolytic Cleavage to Stabilize mRNAs in Arabidopsis. Cell Rep. 2018,25:1146-1157.e1143.

5.	Kohnen MV, Schmid-Siegert E, Trevisan M, Petrolati LA, Sénéchal F, Müller-Moulé P, Maloof J, Xenarios I, Fankhauser C. Neighbor Detection Induces Organ-Specific Transcriptomes, Revealing Patterns Underlying Hypocotyl-Specific Growth. Plant Cell. 2016,28:2889-2904.

6.	Jia J, Lu W, Liu B, Fang H, Yu Y, Mo W, Zhang H, Jin X, Shu Y, Long Y, et al. An atlas of plant full-length RNA reveals tissue-specific and monocots-dicots conserved regulation of poly(A) tail length. Nat Plants. 2021,8:1118-1126.

7.	Courel M, Clément Y, Bossevain C, Foretek D, Cruchez OV, Yi Z, Benard M, Benassy MN, Kress M, Vindry C, et al. GC content shapes mRNA storage and decay in human cells. Elife. 2019,8:e49708.

8.	Ma Z, Zhang X. Actions of plant Argonautes: predictable or unpredictable? Curr Opin Plant Biol. 2018,45:59-67.

9.	Bernardes WS, Menossi M. Plant 3' Regulatory Regions From mRNA-Encoding Genes and Their Uses to Modulate Expression. Front Plant Sci. 2020,11:1252.

10.	Mitschka S, Mayr C. Context-specific regulation and function of mRNA alternative polyadenylation. Nat Rev Mol Cell Biol. 2022,23:779-796.

11.	Alfonso-Gonzalez C, Legnini I, Holec S, Arrigoni L, Ozbulut HC, Mateos F, Koppstein D, Rybak-Wolf A, Bonisch U, Rajewsky N, et al. Sites of transcription initiation drive mRNA isoform selection. Cell. 2023,186:2438-2455.e2422.

12.	Wang Z, Ma Z, Castillo-Gonzalez C, Sun D, Li Y, Yu B, Zhao B, Li P, Zhang X. SWI2/SNF2 ATPase CHR2 remodels pri-miRNAs via Serrate to impede miRNA production. Nature. 2018,557:516-521.

13.	Mori M, Triboulet R, Mohseni M, Schlegelmilch K, Shrestha K, Camargo FD, Gregory RI. Hippo signaling regulates microprocessor and links cell-density-dependent miRNA biogenesis to cancer. Cell. 2014,156:893-906.

14.	Shan L, Xu G, Yao RW, Luan PF, Huang Y, Zhang PH, Pan YH, Zhang L, Gao X, Li Y, et al. Nucleolar URB1 ensures 3' ETS rRNA removal to prevent exosome surveillance. Nature. 2023,615:526-534.

15.	Ding Y, Tang Y, Kwok CK, Zhang Y, Bevilacqua PC, Assmann SM. In vivo genome-wide profiling of RNA secondary structure reveals novel regulatory features. Nature. 2014,505:696-700.

16.	Zubradt M, Gupta P, Persad S, Lambowitz AM, Weissman JS, Rouskin S. DMS-MaPseq for genome-wide or targeted RNA structure probing in vivo. Nat Methods. 2017,14:75-82.

**Second round of review**

**Reviewer 1**

The extensive revision of MS has strengthened this complex work and addressed the majority of my questions. However, some minor questions should be addressed to polish the MS.

1. To enhance the clarity, the authors should annotate the miRNA159/159* in Fig .1a and 1b, to distinguish pri-mi159a-T3 and pri-mi159a-T4.

2. The authors should define all elements of box plots (e.g. minima, maxima, centre, whiskers and percentile) in the legends. This applies to Fig. 1a, 1b, 2a, 2c, 2d, 2e, 2f,4c, 4e, 4f.

3. To enhance the clarity, the authors should add detailed lens information for LUC pictures in Fig .1 a and c, Fig .1b (Line 1203).

4. To improve the readability, the authors should consider erasing the 3, 4, and 5 h in Fig .3b, and 3c to make it easier for readers to differentiate the individual groups.

5. To enhance the clarity, the authors should add the detailed time for the upper and lower panels of Fig. 6a.

6. Line 161, It is not clear why the authors perform 3’RACE experiments here. To complement the logical transition, the authors should briefly introduce 3’RACE add the rationale behind conducting 3’RACE experiments. Moreover, the full name of RACE is missing in the main text of manuscript.

7. Line 243, the citation of sliding window methods is missing.

8. Line 369-370, it is not clear how this conclusion is reached. Reference 32 showed that the RSS structure of global protein-coding transcripts rather than genes involved in the specific terms changed upon salt stress condition. The authors should rephrase “possibly due to” as “indicating” and remove reference 32.

9. Line 375-376, the author should provide detailed information of high-Gini and low-Gini genes in rice and human (the percentile of High- and low-Gini genes and exact numbers).

10. Line 453-455, it is not clear how this conclusion is reached. Three genes are far away from representing any general rule. Please consider removing the sentence “Notably, all these three low-Gini genes are involved in stress-related pathways, which further implies that genes with poorly structured 3’ UTRs are associated with stress responses”.

**Reviewer 2**

The authors have adequately addressed my previous review, and I am pleased to recommend its publication in Genome Biology.

**Reviewer 3**

I am satisfied with the revisions and explanations that the authors have incorporated into the revised manuscript and in their responses to the reviewers. The authors have addressed all of my concerns and have also implemented the recommendation to improve the clarity of the manuscript. Therefore, I recommend the manuscript by Zhang et al. titled "Structured 3' UTRs Destabilize mRNAs in Plants" for publication in Genome Biology.

**Authors Response**

**Point-by-point responses to the reviewers’ comments:**

Reviewer reports:

Reviewer #1:

The extensive revision of MS has strengthened this complex work and addressed the majority of my questions. However, some minor questions should be addressed to polish the MS.

1. To enhance the clarity, the authors should annotate the miRNA159/159* in Fig .1a and 1b, to distinguish pri-mi159a-T3 and pri-mi159a-T4.

**Response: Thanks. We revised the figure and included a sentence in the figure legend: “Be noted that pri-miR159a-T4, but not pri-miR159a-T3 contained the miR159/159* duplex (labeled in green).”.**

2. The authors should define all elements of box plots (e.g. minima, maxima, centre, whiskers and percentile) in the legends. This applies to Fig. 1a, 1b, 2a, 2c, 2d, 2e, 2f,4c, 4e, 4f.

**Response: We revised the detailed description in the figure legends.**

3. To enhance the clarity, the authors should add detailed lens information for LUC pictures in Fig .1 a and c, Fig .1b (Line 1203).

**Response: Thanks. We now added lens information in the figure legend of Fig. 1: “Be noted that LUC pictures in (a) and (c) were taken under a CCD camera (Olympus DP70) different from the one used in (b) (Schneider Kreuznach), with each experiment having its own CK (*P_CHR2_-LUC-3’ UTR*) lines.”. In addition, we also added the lens information in the “Methods” section.**

4. To improve the readability, the authors should consider erasing the 3, 4, and 5 h in Fig .3b, and 3c to make it easier for readers to differentiate the individual groups.

**Response: Thanks for the comment. We thought that deletion of the labels of 3, 4, and 5 h time points might impact the visual clarity and interpretation of our data, therefore, we think that we keep these time points.**

5. To enhance the clarity, the authors should add the detailed time for the upper and lower panels of Fig. 6a.

**Response: Thanks for the comment. We added the detailed days information in Fig. 6a.**

6. Line 161, It is not clear why the authors perform 3’RACE experiments here. To complement the logical transition, the authors should briefly introduce 3’RACE add the rationale behind conducting 3’RACE experiments. Moreover, the full name of RACE is missing in the main text of manuscript.

**Response: Thanks for the comment. We have added one sentence in the main text: “To test this, we first determined the poly(A) site for the *LUC* transcript of *P_CHR2_-LUC-pri-miR159a-3’ UTR* by performing 3’ rapid amplification of cDNA ends (3’ RACE).**

7. Line 243, the citation of sliding window methods is missing.

**Response: Added now.**

Zubradt M, Gupta P, Persad S, Lambowitz AM, Weissman JS, Rouskin S. DMS-MaPseq for genome-wide or targeted RNA structure probing in vivo. Nat Methods. 2017,14:75-82.

8. Line 369-370, it is not clear how this conclusion is reached. Reference 32 showed that the RSS structure of global protein-coding transcripts rather than genes involved in the specific terms changed upon salt stress condition. The authors should rephrase “possibly due to” as “indicating” and remove reference 32.

**Response: Thanks for the comment. We revised the description.**

9. Line 375-376, the author should provide detailed information of high-Gini and low-Gini genes in rice and human (the percentile of High- and low-Gini genes and exact numbers).

**Response: We thank the reviewer for this comment. We added the following sentences: “Likewise, we determined the genes with the top 10% and the bottom 10% Gini indexes as the high-Gini and low-Gini genes in rice, reflecting 1247 (all have Gini index value of 1) and 129 genes, respectively. Similarly, we obtained 129 genes for the high-Gini and low-Gini ones each for human. Interestingly, …”.**

10. Line 453-455, it is not clear how this conclusion is reached. Three genes are far away from representing any general rule. Please consider removing the sentence “Notably, all these three low-Gini genes are involved in stress-related pathways, which further implies that genes with poorly structured 3’ UTRs are associated with stress responses”.

**Response: Thanks for the comment.**

**The conclusion is drawn from the GO enrichment analysis of the high-Gini and low-Gini genes from *Arabidopsis*, as depicted in Fig. 4d. The results revealed that low-Gini genes are more likely to be involved in stress-related biological processes.**

**In Fig. 5c, the three genes are randomly selected representative transcripts with high and low Gini index values, respectively. We found that all these three low-Gini genes are involved in stress-related pathways. This result further supports the idea that genes with poorly structured 3’ UTRs are associated with stress responses, aligning with our GO enrichment analysis results in Fig. 4d.**
